# Nasal Delivery of D-Penicillamine Hydrogel Upregulates a Disintegrin and Metalloprotease 10 Expression via Melatonin Receptor 1 in Alzheimer’s Disease Models

**DOI:** 10.3389/fnagi.2021.660249

**Published:** 2021-04-15

**Authors:** Manli Zhong, Hejia Kou, Pu Zhao, Wei Zheng, He Xu, Xiaoyu Zhang, Wang Lan, Chuang Guo, Tao Wang, Feng Guo, Zhanyou Wang, Huiling Gao

**Affiliations:** ^1^College of Life and Health Sciences, Northeastern University, Shenyang, China; ^2^Department of Histology and Embryology, School of Basic Medical Sciences, China Medical University, Shenyang, China; ^3^Department of Histology and Embryology, School of Medicine, Shenzhen University, Shenzhen, China; ^4^Department of Pharmaceutical Toxicology, School of Pharmaceutical Science, China Medical University, Shenyang, China; ^5^Institute of Health Sciences, Key Laboratory of Medical Cell Biology of Ministry of Education, China Medical University, Shenyang, China

**Keywords:** Alzheimer’s disease, ADAM10, amyloid-β peptide, D-penicillamine, melatonin receptor 1

## Abstract

Alzheimer’s disease (AD) is a type of neurodegenerative disease that is associated with the accumulation of amyloid plaques. Increasing non-amyloidogenic processing and/or manipulating amyloid precursor protein signaling could reduce AD amyloid pathology and cognitive impairment. D-penicillamine (D-Pen) is a water-soluble metal chelator and can reduce the aggregation of amyloid-β (Aβ) with metals *in vitro*. However, the potential mechanism of D-Pen for treating neurodegenerative disorders remains unexplored. In here, a novel type of chitosan-based hydrogel to carry D-Pen was designed and the D-Pen-CS/β-glycerophosphate hydrogel were characterized by scanning electron microscopy and HPLC. Behavior tests investigated the learning and memory levels of APP/PS1 mice treated through the D-Pen hydrogel nasal delivery. *In vivo* and *in vitro* findings showed that nasal delivery of D-Pen-CS/β-GP hydrogel had properly chelated metal ions that reduced Aβ deposition. Furthermore, D-Pen mainly regulated A disintegrin and metalloprotease 10 (ADAM10) expression via melatonin receptor 1 (MTNR1α) and the downstream PKA/ERK/CREB pathway. The present data demonstrated D-Pen significantly improved the cognitive ability of APP/PS1 mice and reduced Aβ generation through activating ADAM10 and accelerating non-amyloidogenic processing. Hence, these findings indicate the potential of D-Pen as a promising agent for treating AD.

## Introduction

Alzheimer’s disease (AD) is a progressive neurodegenerative disease with a substantial medical and socioeconomic impact worldwide. Major pathological hallmarks of AD are senile plaques (SPs), composed of self-polymerized amyloid-β peptide (Aβ), and neurofibrillary tangles (NFTs) of hyperphosphorylated tau proteins (Selkoe, 1991; Hardy and Higgins, 1992). In accordance with the metal hypothesis of AD, Aβ deposition, and tau hyperphosphorylation are aggravated by metal ions, thus promoting the development of AD (Liu et al., 2011; Zhang et al., 2019; Ejaz et al., 2020; Singh et al., 2020; Spotorno et al., 2020). The “metal hypothesis” describes the unbalanced theory of the level and location of metal ions in the pathogenesis of AD (Yang et al., 2019; Patel and Aschner, 2021). The combination of metal and Aβ will affect the conversion of peptides and promote their aggregation into fibrils, which eventually form plaques (Bush and Tanzi, 2008). The unbalance of metal ion concentration could destroy the normal activity of enzymes that regulate the cleavage of APP, leading to excessive Aβ formation (Peron et al., 2018). Moreover, the redox activity of metal ions can induce oxidative stress by triggering the production of reactive oxygen species (ROS; Guo et al., 2015; Liu et al., 2019). Metal dyshomeostasis can induce neurodegeneration via inhibiting self-polyubiquitination reactions (Atrian-Blasco et al., 2017) or neuron damage via modulating autophagy (Zhang et al., 2016; Liu et al., 2018).

Recently, metal dyshomeostasis has been attracted more attention as a causative agent for AD. A large number of key studies have shown that several new molecules rebalancing the concentration of metal ions in the brain can inhibit Aβ aggregation, break down amyloid plaques and slow down AD-related cognitive function decline in AD, many of which have also advanced through clinical trials (Ritchie et al., 2003; Lannfelt et al., 2008; Faux et al., 2010). D-penicillamine (D-Pen) is a beta dimethyl analog of the amino acid cysteine, chelating with heavy metals to increase their urinary excretion in patients with Wilson’s disease, rheumatoid arthritis, and cystinuria (Squitti et al., 2018). D-Pen mainly chelates excess copper (I) by forming stable complexes via thiol groups, allowing for renal excretion of copper (Delangle and Mintz, 2012). Recently, some researchers have proposed that the chelating abilities of D-Pen may aid in the treatment of AD. Research has indicated that oral D-Pen administration can reduce copper and zinc superoxide dismutase activity in the blood of patients with AD (Squitti et al., 2002). Additional studies have reported that D-Pen nanoparticles can solubilize copper-Aβ (1-42) aggregates *in vitro* (Cui et al., 2005). However, the chemical structure of D-Pen renders it too hydrophilic to cross the blood–brain barrier (BBB; Cui et al., 2005). In recent decades, researchers have increasingly focused on the potential of nasal drug delivery (Illum, 2000; Casettari and Illum, 2014), which enables drug absorption through circumventing the BBB that avoid initial liver metabolism, and reduce durg side effects. Such delivery systems have received attention for the treatment of chronic neurodegenerative diseases such as Parkinson’s disease and AD (Aderibigbe, 2018; Agrawal et al., 2018; Alexander and Saraf, 2018).

Chitosan (CS) is a polysaccharide originating from crustacean shells and is both biocompatible and recognized as generally safe. When being dropped or sprayed into the nose at 35–37°C, the thermosensitive CS or CS hydrogel transfers to a gel state from the liquid state, thereby decreasing the nasal mucociliary clearance rate and allowing for sustained drug release. The CS system in nasal delivery transports the hydrophilic molecules across the membrane to improve biocompatibility and bioavailability and extend drug retention time (Vllasaliu et al., 2010; Casettari and Illum, 2014; Sarvaiya and Agrawal, 2015). Therefore, we attempted to exploit the characteristics of CS to deliver D-Pen bypass the BBB.

In the present study, we developed a D-Pen-carrying CS/β-glycerophosphate (β-GP) hydrogel and characterized the potential effects of its nasal administration in a mouse model of AD. Our aim was to examine the specific mechanism of action of D-Pen in the treatment of AD.

## Materials and Methods

### Nasal Hydrogel Preparation

Table 1 summarizes the reagent information. The D-Pen-CS/β-GP hydrogel was synthesized according to the method listed in the supporting information (Supplementary Figure 1). D-Pen was first dissolved in acetic acid/sodium acetate (v/v = 1/4). After the solution became clear, 2% (w/w) CS (low molecular weight, 75–85% deacetylated) was added and stirred for approximately 1 h until thoroughly dissolved. Chilled β-glycerophosphate (β-GP) solution (0.5 g/L) was added drop-wise to the CS solution to counterbalance its pH increasing tendency. The final pH of the D-Pen-CS/β-GP hydrogel ranged from 6.9 to 7.2.

**TABLE 1:** List of reagents.

Reagents	Code number	Company
Alexa Fluor^®^ 488-conjugated donkey anti-rabbit IgG	A-21206	Thermo Fisher Scientific
Alexa Fluor^®^ 546-conjugated donkey anti-mouse IgG	A-10036	Thermo Fisher Scientific
Biotinylated goat anti-mouse IgG	E043301-2	Dako
Cell Titer 96^®^ AQ_ueous_ One Solution Reagent	G3582	Promega
Chitosan	448869	Sigma-Aldrich
Cyclic adenosine monophosphate (cAMP) Assay Kit	H164	Jiancheng Biology
D-Penicillamine	P4875	Sigma-Aldrich
GI 254023X	HY-19956	Med Chem Express
H89 2HCl	S1582	Selleck
Human amyloid beta peptide 1–40, Aβ1–40 ELISA Kit	CSB-E08299h	Cusabio
Human β Amyloid (1–42) ELISA High-Sensitive Kit	296-64401	Wako
N-(6-methoxy-8-quinolyl)-*p*-toluenesulfonamide (TSQ)	M688	Thermo Fisher Scientific
Peroxidase AffiniPure Goat Anti-Mouse IgG (H + L)	115-035-003	Jackson Immuno Research
Peroxidase AffiniPure Goat Anti-Rabbit IgG (H + L)	111-035-003	Jackson Immuno Research
Phosphatase inhibitor cocktail	07574-61	Nacalai Tesque
Protease inhibitor cocktail	03969-21	Nacalai Tesque
Protein kinase A (PKA) activity kits	H233-1	Jiancheng Biology
U0126	9903	Cell Signaling Technology
VECTASTAIN^®^ABC KIT	PK-4000	Vector Laboratories
β-GP disodium salt hydrate	D-106347	Aladdin

### Thermo-Sensitive Testing and Characterization of D-Pen-CS/β-GP Hydrogel

The two bottles with D-Pen-CS/β-GP gel and D-Pen liquid were stored at 25 and 37°C, respectively. Then, the CS/β-GP gel and D-Pen-CS/β-GP gel shapes and surface morphology were observed under the SU8010 field emission scanning electron microscopy (Hitachi Technologies Inc., Japan). To characterize the chemical structure of the D-Pen-CS/β-GP solution at 37°C, Data of fourier transform infrared (FT-IR) spectra were obtained using a Nicolet-6700 FT-IR spectrophotometer (Thermo Ltd., United States).

### Animals and Treatment

APPswe/PS1d9 double-transgenic mice (original from Jackson Laboratory, Bar Harbor, ME, United States) and C57BL/6 mice were housed under a light/dark cycle of 8:00/20:00 and controlled temperature (24 ± 1°C) and humidity (50–60%) conditions. This study was conducted in strict accordance with the guidelines of the Animal Ethics Committee of the China Medical University.

APP/PS1 mice (6 months old, *n* = 16) were separated into two equisized groups. The treatment group was administered 2 mg/kg D-Pen-CS/β-GP hydrogel once every other day for 3 months. The control group received CS/β-GP hydrogel alone according to the same schedule. All animals were sacrificed following behavioral testing.

C57BL/6 mice were used for inductively coupled plasma mass spectrometry and primary culture in this experiment.

### High Performance Liquid Chromatography

APP/PS1 mice (6 months old, *n* = 16) were randomly divided into four groups. Aliquots of phosphate-buffered saline (PBS; vehicle), 2 mg/kg D-Pen liquid, 2 mg/kg D-Pen-CS/β-GP hydrogel, or CS/β-GP hydrogel only (without loading D-Pen) were intranasally administered for 2 h, and the whole brain was resected. One milliliter of radio immunoprecipitation assay lysate was added to the mouse brain homogenates and mixed well overnight, then centrifuged at 13,000 rpm for 30 min. The supernatants were separated into a 1.5 mL centrifuge tube and dropped into the ultrafiltration centrifuge tube (3 kDa molecular weight cut-off) to remove the interference of proteins in the tissue. The centrifuged solutions were freeze-dried, concentrated, and then re-dissolved by adding 1 mL of water. A standard stock solution of D-Pen was prepared at 1 mg/mL. The XBridge peptide BEH C18 (4.6 mm × 250 mm, 5 μm) column was selected, and the temperature was maintained at 25°C. The analysis was performed using 0.1% trifluoroacetic acid–acetonitrile at a flow rate of 1 mL/min and ultraviolet detection (Waters model 998; Waters Corp., Milford, MA, United States) at 254 nm. Concentrations of D-Pen were determined from the chromatographic peak areas.

### Behavioral Tests

The Morris Water Maze (MWM) test and nest construction (NC) test were used to assess spatial learning and memory. For the MWM, briefly, the experimental apparatus consisted of a circular plastic pool (100 cm dia. × 40 cm H) and filled with water (23 ± 2°C) colored with non-toxic white paint to obscure the location of a submerged platform. The mice were subjected to a pre-training (visible platform), composed of three trials with intervals of 60 min over 2 days. A limit of 60 s was given for the mice to find the visible platform. Then, the target platform (10 cm × 10 cm) was submerged 1 cm below the water surface and placed at the midpoint of one quadrant. The entire experiment was conducted over a period of 7 days, and mice were given 60 s to find the platform. Twenty-four hours after the last hidden platform test, the hidden platform was removed for the probe trial. The number of times the animal crossed the center of the previous location of the hidden platform within an interval of 60 s was recorded. Swimming was tracked by video SMART v3.0 (Panlab Harvard Apparatus, United States), and the latency, path length, swim speed, and cumulative distance from the platform were recorded. Mean swim latency for each day was evaluated and compared among groups. All analyses were performed by an investigator blind to the experimental conditions.

For the NC test, mice were housed individually in single cages. Ten pieces of paper (5 cm × 5 cm) were introduced into the cage on the first day of testing and observed for 5 days through photographic recording. The quality of the nest was rated along a 5-point scale, as follows (Wang et al., 2018): 1 = no noticeably torn paper; 2 = no noticeably torn paper, with a partially identifiable nest site; 3 = mostly shredded paper but no identifiable nest site; 4 = partially shredded paper, with an identifiable nest; and 5 = all shredded paper, a (near) perfect nest.

### Cell Cultures and Treatments

Mouse neuroblastoma (N)2a cells and N2a cells stably overexpressing APP Swedish mutation (N2a-sw) were donated by Professor Huaxi Xu (College of Medicine, Xiamen University) (Thinakaran et al., 1996; Zhang et al., 2009; Bulloj et al., 2010; Huang et al., 2010). The cells were grown on 10-cm tissue culture dishes in 10 mL Dulbecco’s Modified Eagle Medium (DMEM) supplemented with 10% fetal bovine serum (FBS) and N2a-sw were additionally supplemented with 200 μg/mL G418 (10131035, Invitrogen). After undergoing culture in a FBS-free medium for additional 6 h, the cells were treated with 10 and 25 μM D-Pen in FBS-free medium for another 24 h. After starved with DMEM without FBS, N2a-sw cells were pre-incubated with 10 μM GI 254023X, 5 μM H89, and 10 μM U0126 in each separate experiment for 2 h (Durairajan et al., 2011; Shukla et al., 2015; Wang et al., 2018), and then cells were incubated with 10 μM of D-Pen for an additional 24 h.

### Primary Culture and Aβ Treatment

The meninges were removed and the cerebral cortices of the C57BL/6 neonatal mice (<24 h) were isolated and rinsed with sterile PBS pH 7.4, and finely minced into small pieces. The minced tissue was then incubated with Papain solution (2 mg/mL) for 30 min at 37°C (Nunez, 2008). The cell suspensions were filtered through the cell strainer (500 μm, BD Company, San Diego, CA, United States) and centrifuged at 500 × *g* for 10 min and the cell pellets were suspended in plating medium DMEM with L-glutamine (Sigma Aldrich, S. Louis, MI, United States) + 10% FBS + 100 units/mL penicillin + 0.1 mg/ml of streptomycin on poly-L-lysine-coated plates (Corning Company, Corning, NY, United States) at a density of 5 × 10^6^ cells/mL. After the cells were attached, the medium was changed to Neurobasal Medium and B27 supplement (Thermo Fisher, Waltham, MA, United States). Half of the medium was replaced with fresh medium every 3 days.

Aβ42 oligomers were prepared as described previously (Stine et al., 2011). Briefly, human Aβ (1–42) peptide (Sigma Aldrich) was dissolved in 1,1,1,3,3,3-hexafluoro-2-propanol (Sigma) in a chemical fume hood to obtain 1 mM solution. Oligomers were prepared by diluting 10 mM Aβ dimethyl sulfoxide stock to 0.01 mM with PBS and incubated at 37°C for 24 h. Before being added to neurons, Aβ oligomer preparations were diluted to 2 μM with neuronal medium and used to replace the entire medium. Control cultures were treated with the same concentration of dimethyl sulfoxide. After Aβ oligomer treatment for 24 h, the cells were incubated with fresh medium with or without freshly prepared Aβ oligomers and D-Pen (10 or 25 μM) for an additional 24 h.

### Inductively Coupled Plasma Mass Spectrometry

The cortex of C57BL/6 mice and APP/PS1 mice (D-Pen-CS/β-GP hydrogel group and control group *n* = 8 of each group) were weighted, collected following centrifugation and the N2a-sw cell pellets were collected. Both of these pellets were digested in 90% HNO_3_ at 105°C for 30 min. The samples were then diluted, and the metal content of each diluted solution was detected via inductively coupled plasma mass spectrometry (ICP-MS) using an Agilent Technologies 7500a instrument. Isotopes ^63^Cu, ^56^Fe, and ^66^Zn were chosen for optimal sensitivity of quantification in data acquisition mode (spectral analysis).

### Immunohistochemistry

After behavioral test, the mice in the two experimental groups (*n* = 8 in each group) were anesthetized and half brains were fixed with 4% paraformaldehyde and embedded in paraffin. Briefly, 5-μm coronal sections were treated with a blocking buffer [5% bovine serum albumin (BSA) and 1% normal goat serum] for 1 h and then incubated with mouse monoclonal anti-Aβ antibody (1:500, Table 2) overnight at 4°C. After rinsing, the sections were incubated in biotinylated goat anti-mouse IgG (1:500), and processed with the avidin–biotin–peroxidase (ABC) complex (1:100). Finally, the sections were immersed in 3,3’-diaminobenzidine. Incubation of one section in normal mouse serum (1:100) served as a negative control for non-specific staining.

**TABLE 2:** List of primary antibodies.

Antibody	Resource	Code number	Company
anti -ADAM10^a,b^	rabbit	ab84595	Abcam
anti-Anti-BACE1 [EPR3956]^b^	rabbit	ab108394	Abcam
anti-APP C-terminal^b^	rabbit	A8717	Sigma-Aldrich
anti-BAX^b^	rabbit	A3533	Dako
anti-Bcl-2^b^	mouse	M0887	Dako
anti-COXIV^b^	rabbit	PA5-29992	Thermo Fisher Scientific
anti-CREB (48H2)^b^	rabbit	9197	Cell Signaling Technology
anti-glial fibrillary acidic protein (GFAP)^a, b^	rabbit	Z0334	Dako
anti-Human sAPPα (2B3)^*b*^	mouse	11088	IBL
anti-Human sAPPβ-sw (6A1)^*b*^	mouse	10321	IBL
anti-MAP-2^*a*^	mouse	M4403	Sigma-Aldrich
anti-Melatonin Receptor 1α (MTNR1α)^b^	rabbit	ab203038	Abcam
anti-Melatonin Receptor 1β (MTNR1β)^b^	rabbit	ab203346	Abcam
anti-p44/42 MAPK (Erk1/2)^b^	rabbit	9102	Cell Signaling Technology
anti-Phospho-CREB (Ser133) (87G3)^®b^	rabbit	9198	Cell Signaling Technology
anti-Phospho-p44/42 MAPK (Erk1/2) (Thr202/Tyr204)^®^^b^	rabbit	9101	Cell Signaling Technology
anti-phospho-PKA C (Thr197) (D45D3)^b^	rabbit	5661	Cell Signaling Technology
anti-PKA C-α^b^	rabbit	4782	Cell Signaling Technology
anti-Presenilin 1 (D39D1)^b^	rabbit	5643	Cell Signaling Technology
anti-Presenilin 2 (D30G3)^b^	rabbit	9979	Cell Signaling Technology
anti-β-Actin^*b*^	mouse	A1978	Sigma-Aldrich
anti-β-Amyloid^*a*^	mouse	A8354	Sigma-Aldrich

*^a^The antibody is used for immunochemistry or immunofluorescence staining. ^b^The antibody is used for Western blot.*

Using unbiased stereological method (West, 1999), labeled SPs counted on 10 sections of each mouse were observed under a Lica DMI4000 B microscope using a 4× objective and images acquired by LAS V4.1 system. The sizes of Aβ-positive plaques in the cortex were measured by NIH ImageJ software. The total number of Aβ-positive plaques in the cortex was estimated using the optical fractionator formula = 1/ssf (slice sampling fraction) × 1/asf (area sampling fraction) × 1/tsf (thickness sampling fraction) × Σ (number of objects counted).

### Immunofluorescence Staining and Confocal Laser Scanning Microscopy

Paraffin sections of the cortex were deparaffinized then incubated with a mixture of rabbit anti-glial fibrillary acidic protein (anti-GFAP) antibody (1:500) overnight at 4°C. Primary neurons cultured on coverslips were fixed with 4% PFA in PBS. Fixed primary cultured cells were blocked with a blocking buffer and incubated with a mixture of rabbit anti-ADAM10 antibody (1:500) and MAP-2 monoclonal antibody (1:500, Sigma Aldrich) overnight at 4°C. After rinsing, sections were incubated with Alexa Fluor^®^ 488-conjugated goat anti-rabbit immunoglobulin (IgG) (1:1,000) and Alexa Fluor^®^ 546-conjugated goat anti-mouse IgG (1:1,000) for 2 h at room temperature. Laser scanning confocal microscopy was performed at 400× magnification to measure the average fluorescence intensity (Leica TCS SP8). The antibodies’ details used for Immunofluorescence staining were in Table 2.

### Western Blot

The cortex (*n* = 8 in each group) and the pellets of cells treated with different formulations were homogenized in ice-cold lysis buffer with protease inhibitor cocktail and phosphatase inhibitor cocktail (1:100 each). After centrifugation (12,000 × *g*, 30 min, 4°C), protein levels were determined using a BCA protein assay kit. Proteins (10 μg) were separated onto 4–12% SDS polyacrylamide gels and transferred onto polyvinylidene fluoride (PVDF) membranes. The membranes were incubated in 5% BSA for 1 h and subsequently incubated with primary antibodies (Table 2) overnight at 4°C. Following treatment with horseradish peroxidase-conjugated anti-rabbit or mouse secondary antibodies (1:5,000, 2 h), the specific protein bands were examined using an enhanced chemiluminescence kit (ECL). Protein intensities were semi-quantitatively analyzed using the NIH ImageJ software.

We examined sAPPα and sAPPβ secretions as previously described (Shukla et al., 2015). The cells (about 5 × 10^5^/dish) were treated with or without D-Pen (10, 25 μM) and/or GI 254023X (10 μM) after reaching 80% confluence in 60-mm dishes. The media (DMEM, 50 μL) were directly subjected to Western blot analysis.

### Enzyme-Linked Immunosorbent Assay and Enzymatic Spectrophotometric Methods

Using Human β Amyloid (1-42) enzyme-linked immunosorbent assay (ELISA) High-Sensitive Kit and Human β Amyloid (1–40) ELISA Kit, Aβ1–42, and Aβ1–40 levels were determined in accordance with the manufacturers’ instructions. The activity of protein kinase A (PKA) and cyclic AMP response element binding protein (cAMP) in N2a-sw cells was detected via colorimetry, in accordance with the instructions for the corresponding kits.

### Cell Viability

Cell Titer 96^®^ AQ_ueous_ One Solution Reagent was conducted to evaluate the effect of D-Pen on the viability of N2a cells. 1 × 10^4^ N2a-sw cells per well were seeded in 96-well plates and treated with different concentrations of D-Pen (0.5, 1, 2, 5, 10, 25, 50 μM) for 24 h at 37°C. Subsequently, the cells in 100 μL of culture medium was added 20 μL of Cell Titer 96^®^ AQ_ueous_ One Solution Reagent in each well and further incubated for 4 h. The absorbance was measured at 490 nm in a microplate reader (Bio-Rad, Laboratories, Inc., Hercules, CA, United States).

### Zinc Staining

N-(6-methoxy-8-quinolyl)-p-toluenesulfonamide (TSQ) staining was performed as previously described (Frederickson et al., 1992; Varea et al., 2001). Briefly, N2a-sw cells were cultured in 24-well plates after treatment with D-Pen and immersed in 4.5 μM TSQ solution for 1 min. The zinc reactions were imaged using a fluorescence microscope (Nikon, Ds-Ri1).

### siRNA Transfection and Real-Time PCR

The siRNA duplexes against melatonin receptor 1 (MTNR1 α) was designed by us and synthesized by Genepharma with the following sequences. MTNR1α: GGAUCUACUCCUGUACCUUTT (sense) and AAGGUACA GGAGUAGAUCCTT (anti-sense). N2a-sw cells were seeded on 6-well plates and transfected with 50 nM scrambled-siRNA or targeted-siRNA using Lipofectamine RNAiMAX Reagent (13778030, Invitrogen) according to the manufacture’s specifications. Cells were further cultured for 48 h before being treated with D-Pen (10 μM) for 24 h. The knockdown efficiency was validated by real-time PCR.

The total RNA was isolated from N2a-sw cells with scrambled-siRNA or MTNR1α-siRNA using TRIzol reagents (Invitrogen) according to the manufacturer’s instructions. Five-hundred nanogram of template RNA was reverse transcribed to cDNA using Reverse Trasncription System (Promega, A5001) and the obtained cDNA was used to the subsequent PCR reactions. All PCR reactions were performed in a total volume of 20 μL: DNA polymerase activation at 95°C for 2 min, and about 50 cycles for denaturing at 95°C 10 s, annealing at 55°C 10 s and extension at 72°C 30 s. The following PCR primers were used: MTNR1α: forward, GGACCATGAAGGGCAATGTCAG and reverse CCTGAGTTCCTGAGCTTCTTG. GAPDH: forward, TGCGACTTCAACAGCAACTC and reverse, GGTCT GGGATGGAAATTGTG. The mRNA expression was calculated using ^ΔΔ^ Ct (threshold cycle, Ct) values normalized to GAPDH.

### Statistical Analyses

All the experiments and analyses were conducted with the experimenter blind to drug treatment. For *in vitro* experiments, all statistical values were from at least three independent experiments. Descriptive statistics were expressed as the mean ± the standard error of the mean (SEM). Comparisons were performed using one-way analysis of variance (ANOVA), followed by Tukey’s *post hoc* test or two-tailed Student’s *t*-tests. The statistical significance level was set to ^∗^*p* < 0.05 vs untreated control group.

## Results

### Characterization of D-Pen-CS/β-GP

**FIGURE 1:**
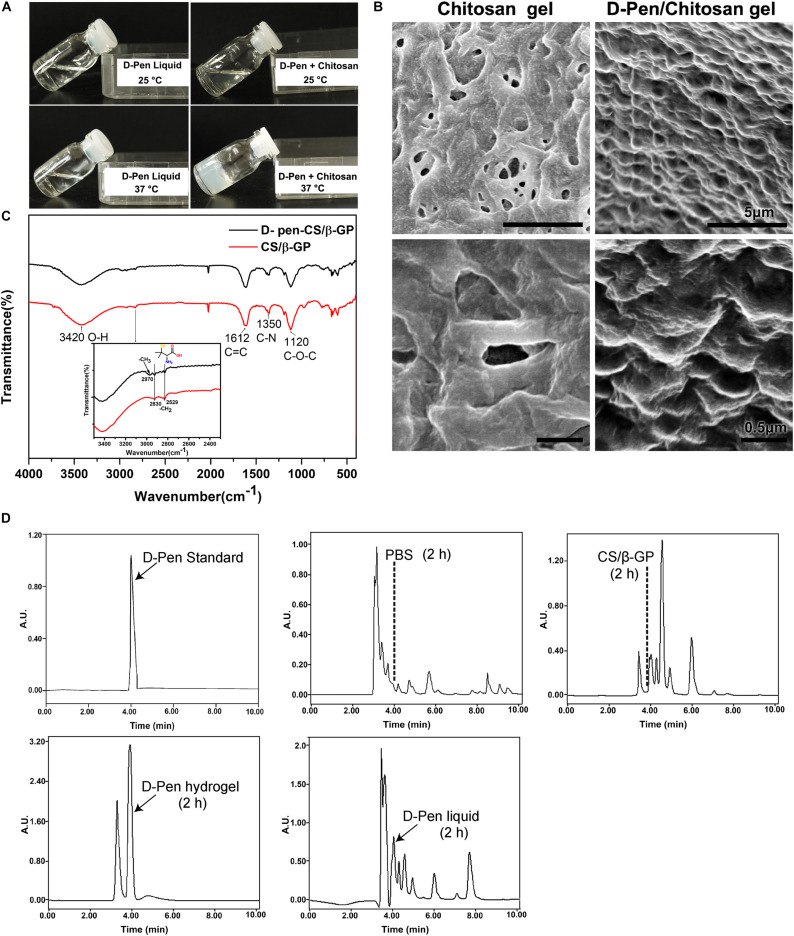
Preparation and characterization of D-Pen-CS/β-GP. **(A)** The D-Pen hydrogel and water solution were examined at different temperatures to analyze their thermosensitivity. **(B)** Surface characterizations of the CS/β-GP and D-Pen-CS/β-GP gels by scanning electron micrography. **(C)** FT-IR spectra of the CS/β-GP and D-Pen-CS/β-GP gels. **(D)** HPLC spectra detected the peaks of D-Pen standard at 4 min and also detected the peaks of D-Pen in the brain after 2 h of PBS, CS/β-GP, D-Pen hydrogel, and D-Pen liquid treatment, respectively.

The hydrophilic nature of D-Pen limits its applications in the treatment of central nervous system diseases. To circumvent this restriction, we designed a D-Pen-CS/β-GP hydrogel and we performed thermo-sensitivity analyses by subjecting the D-Pen hydrogel and water solution to different temperatures. At room temperature (approximately 25°C), both the hydrogel and water solution existed as clear and transparent liquids, and the hydrogel could be maintained in the liquid state for >6 h. When the temperature was increased to 37°C, D-Pen-CS/β-GP transformed into an opaque, white gel (Figure 1A). On scanning electron microscopy, we observed that the CS/β-GP gel exhibited an irregular porous structure, while the CS/β-GP gel loaded with D-Pen (Figure 1B) was rougher and more textured.

As shown in Figure 1C, the absorption bands of CS/β-GP gel at 1,612 and 1,350 cm^–1^ indicated the assignation to the N–H bending of the deacetylated amine (-NH_2_) and the carbonyl stretching of a non-deacetylated amide (NHC=OCH_3_; amide I band), respectively. After the chemical reaction, the amine peak for D-Pen-CS/β-GP gel at 1,350 cm^–1^ was weakened, and such weakening was due to the loading of the D-Pen-CS/β-GP gel. In addition, the CS/β-GP gel contained a –CH_2_ group only, whereas D-Pen contained both –CH_2_ and –CH_3_ groups. Thus, a new absorption band for D-Pen-CS/β-GP gel was observed at 2,970 cm^–1^. The absorption bands at 2,970 cm^–1^ represented the -CH_3_ stretching vibrations of D-Pen. The characteristic absorption peaks of CS/β-GP gel and D-Pen-CS/β-GP gel were also detected in the spectra. The above results indicated that D-Pen was successfully loaded.

Compared with the PBS group, the peak of the D-Pen-CS/β-GP group obviously appeared at 4 min, which indicated that D-Pen was successfully delivered to the brain of AD mice. After 2 h of treatment, the concentration of D-Pen in the D-Pen-CS/β-GP gel group was higher than that in the D-Pen liquid group, while no effective peaks were observed in the PBS group and the CS/β-GP group at 4 min (Figure 1D). Immunohistochemistry results revealed significant differences in Aβ-positive plaques number and shape among the four groups of APP/PS1 mice (Supplementary Figure 2A). In the D-Pen hydrogel group, the number of Aβ-positive plaques decreased, and the plaques were smaller than those in the other three groups (Supplementary Figures 2B,C). The results indicated the effect was significantly better in the D-Pen gel group than in the liquid group, and there was no significant difference between the PBS and CS/β-GP gel groups.

### D-Pen-CS/β-GP Chelation and Cytotoxicity *in vitro* and *in vivo*

**FIGURE 2:**
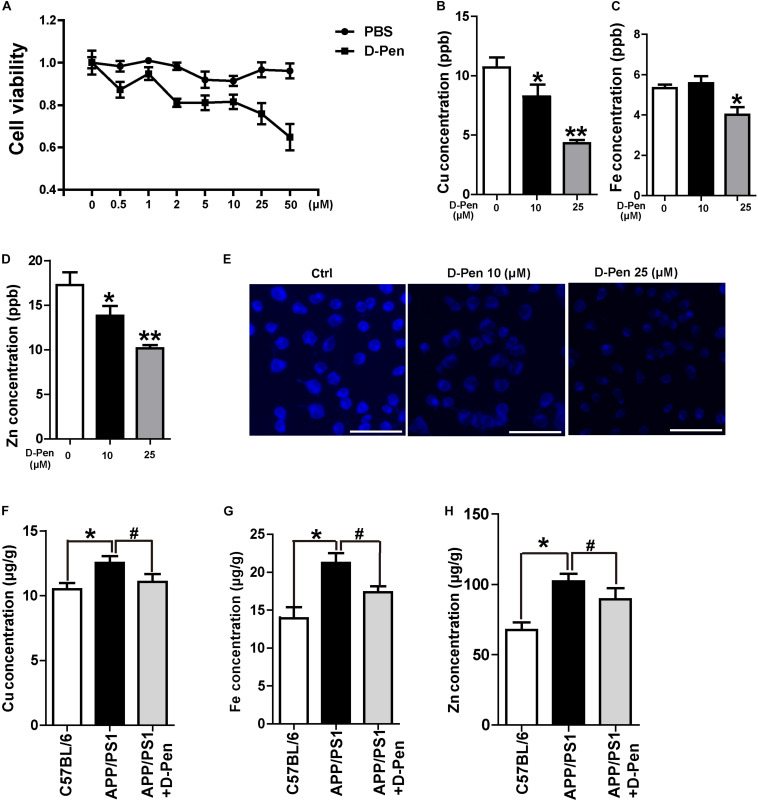
Chelation and cytotoxicity of the D-Pen *in vitro*. **(A)** Cell viability of N2a-sw cells incubated with different concentrations of D-Pen for 24 h. All values are presented as the mean ± standard error of the mean (SEM) of at least three independent experiments. **(B)** ICP-MS revealed that the copper concentrations in N2a-sw cells with medium containing 10 or 25 μM D-Pen. **(C)** ICP-MS revealed that the iron concentrations in N2a-sw cells with medium containing 10 or 25 μM D-Pen. **(D)** ICP-MS revealed zinc concentrations in N2a-sw cells with medium containing 10 or 25 μM D-Pen. All values at least three independent experiments are presented as the mean ± standard error of the mean (SEM) by one-way analysis. *p < 0.05, **p < 0.01 compared to N2a-sw cells. **(E)** TSQ staining verified that zinc fluorescence intensity decreased in N2a-sw cells with medium containing 10 or 25 μM D-Pen. Bar = 30 μm. **(F–H)** ICP-MS revealed the concentrations of copper, iron, and zinc in the cerebral cortex of C57BL/6 mice, APP/PS1 mice, and D-Pen hydrogel group of APP/PS1 mice. All values are presented as the mean ± SEM (*n* = 8/each group) by one-way analysis. *p < 0.05 vs C57BL/6 mice, ^#^*p* < 0.05 vs APP/PS1 mice.

Firstly, N2a-sw cells were cultured with media containing different concentrations of D-Pen solution for 24 h to assess the effect of D-Pen on cell viability. As is shown in Figure 2A, cell viability was inversely related to different D-Pen concentrations. About 10 and 25 μM D-Pen were selected as the administration concentration to N2a-sw cells for 24 h, which lead to a cell viability of 90 and 78%, separately. ICP-MS was used to detect the metal concentrations in cells. Our results indicated that copper was chelated by D-Pen as its cellular concentrations decreased when being treated with 10 and 25 μM D-Pen (Figure 2B), 25 μM D-Pen also reduced the content of iron in N2a-sw cells (Figure 2C). ICP-MS revealed that zinc concentrations also decreased, and TSQ staining confirmed that zinc fluorescence intensity had weakened in the cell media containing 10 and 25 μM D-Pen (Figures 2D,E).

Consistent with previous results (Bush, 2003; Roberts et al., 2012), the iron, zinc, and copper contents in the cerebral cortex of APP/PS1 mice were higher than those in the cerebral cortex of C57BL/6 mice. As an effective metal chelator, D-Pen could chelate the contents of copper, iron, and zinc ions in the brains of AD mice. Although it failed to restore the metal content to normal levels, there was no significant difference in the metal contents compared with those in the cerebral cortex of C57BL/6 mice (Figures 2F–H).

### D-Pen-CS/β-GP Improved Cognitive Decline in APP/PS1 Transgenic Mice

**FIGURE 3:**
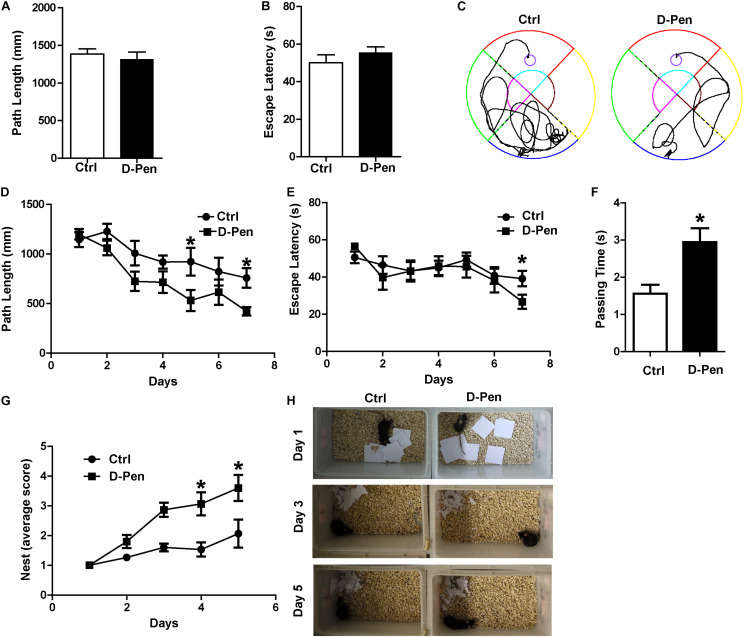
D-Pen-CS/β-GP treatment ameliorated cognitive deficits in APP/PS1 mice. The path length **(A)** and escape latency **(B)** did not significantly changed on the visible platform trail. **(C)** Representative escape routes on the 5 day of the hidden platform test were shown. **(D)** Swimming distances and **(E)** escape latency over 7 days of training and memory assessment. **(F)** A spatial probe test was conducted 24 h after completion of the navigation test, and the number of platform crossings was assessed. **(G,H)** Mice were individually housed in cages with 10 square pieces of paper. The changes in the mouse bite paper were recorded for five consecutive days by photographic recording. All values are presented as the mean ± standard error of the mean; *n* = 8 mice per group. *p < 0.05, compared to the vehicle control group (two-tailed Student’s *t*-test).

In order to examine the therapeutic effect of D-Pen hydrogel, we firstly investigated the nasal administration of D-Pen-CS/β-GP hydrogel exerted neuroprotective effects against AD by evaluating spatial learning and memory abilities of APP/PS1 mice. In the visible platform test, we did not find significant differences in path length (Figure 3A) or escape latency (Figure 3B) between D-Pen treatment and the control groups, indicating that D-Pen treatment did not affect vision and motility in this animal model. In the hidden platform tests after 3 months of intranasal delivery, as training days increased, the distance and time taken for the mice to find the platform in the two groups gradually shortened. APP/PS1 mice treated with D-Pen-CS/β-GP hydrogel exhibited clear improvements in learning ability (Figure 3C). The path length of the control group was about 882.42 mm on day 4, and was about 744.64 mm on day 6. Following D-Pen-CS/β-GP hydrogel treatment, path length was also lower than that observed in control mice on day 4 (*p* < 0.05) and day 6 (*p* < 0.05, Figure 3D). Furthermore, the escape latency of control group on day 6 was about 44.85 ms. D-Pen-CS/β-GP hydrogel treatment significantly decreased the escape latency relative to that in controls on day 6 (*p* < 0.05, Figure 3E). It also significantly increased the incidence of platform crossings (*p* < 0.05, Figure 3F) compared with the controls. For the non-cognitive behavioral examination, APP/PS1 mice and mice in D-Pen treatment group had the ability to build nests. The average nesting scores of APP/PS1 mice is 1–2 points. The nesting scores of the APP/PS1 mice significantly improved after D-Pen treatment (*p* < 0.05 for days 3 and 4, Figures 3G,H). These results showed that D-Pen-CS/β-GP hydrogel treatment ameliorated the memory deficits in APP/PS1 mice.

### D-Pen-CS/β-GP Treatment Enhanced Non-amyloidogenic Processing *in vivo*

**FIGURE 4:**
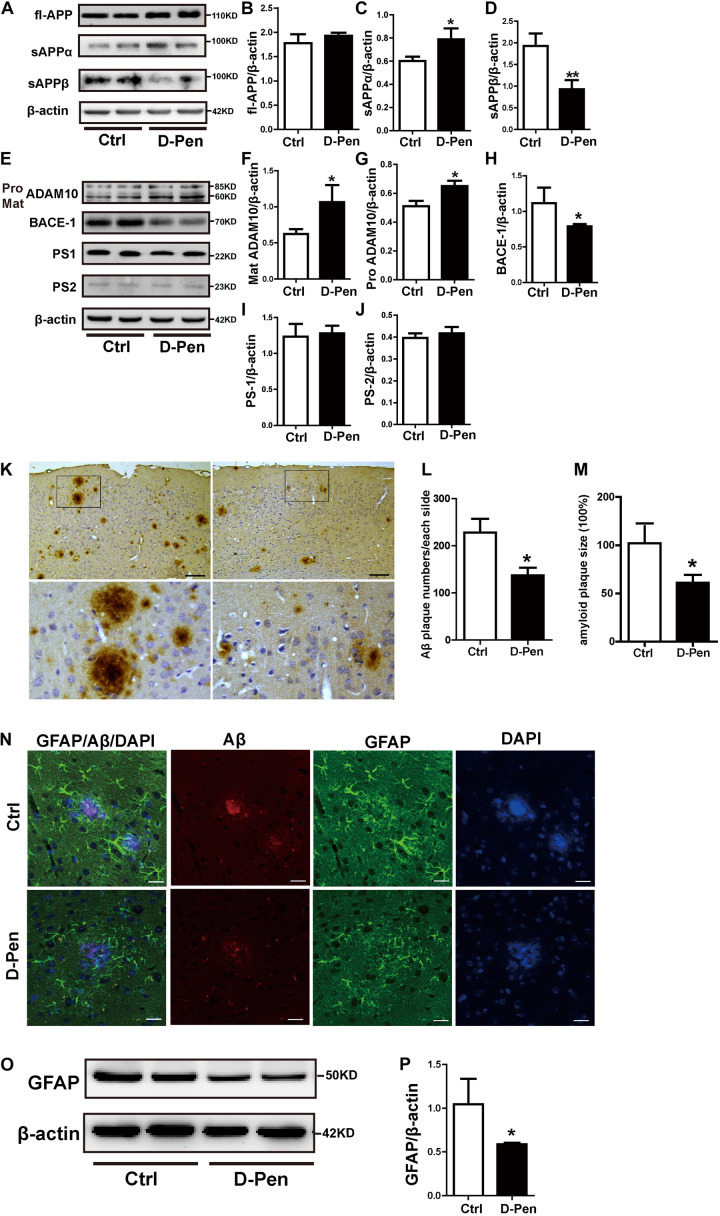
D-Pen-CS/β-GP regulated APP processing and reduced the deposition of Aβ in the cerebral cortex of APP/PS1 mice. **(A)** Western blot analysis revealed the protein bands of APP, sAPPα, and sAPPβ in D-Pen-CS/β-GP hydrogel-treated and control mouse brains. **(B–D)** Gray value analysis of fl-APP, sAPPα, and sAPPβ protein levels in the mouse cerebral cortex. **(E)** Western blot analysis showed the protein bands of ADAM10, BACE1, PS1, and PS2 in D-Pen-CS/β-GP hydrogel-treated and control mouse brains. **(F–J)** The gray value levels of APP-processing proteins, including ADAM10, BACE1, PS1, and PS2, were analyzed with ImageJ software. β-actin was used as an internal control. All values are presented as the mean ± standard error of the mean (SEM) (*n* = 8). *p < 0.05, **p < 0.01 compared to the vehicle control group (two-tailed Student’s *t*-test). Aβ immunohistochemistry **(K)** revealed SPs in the cerebral cortex of D-Pen and control mice. Bar = 30 μm. Representative Aβ plaques in coronal brain sections are shown in the black frame. Plaque numbers and amyloid plaques size **(L,M)** in coronal sections were analyzed using ImageJ (*n* = 8). Representative photomicrographs of GFAP/Aβ fluorescent colocalization staining in the cerebral cortex (*n* = 8). **(N)** GFAP-immunopositive astrocytes were observed around Aβ-positive plaques in control and D-Pen mice (scale bar = 20 μm). Quantitative analysis revealed that GFAP **(O,P)** levels were markedly reduced following D-Pen treatment. All values are presented as the mean ± standard error of the mean (SEM). *p < 0.05 compared to the vehicle control group (two-tailed Student’s *t*-test).

The levels of APP and enzymes involved in APP cleavage affected by D-Pen were examined via Western blot analysis (Figures 4A–D). No significant differences in protein levels of fl-APP were observed between the vehicle and D-Pen groups (*p* > 0.05, Figures 4A,B). However, there was a modest decrease in the protein level of sAPPβ and a significant increase in sAPPα expression in the D-Pen group, when compared with the levels in control APP/PS1 mice (Figures 4C,D). Furthermore, D-Pen significantly increased the expression levels of ADAM10 containing pro-ADAM10 and mature-ADAM10 (*p* < 0.05, Figures 4E–G) and reduced BACE1 protein levels in APP/PS1 mice (*p* < 0.05, Figures 4E,H). However, no specific differences in PS1 or PS2 expression were observed between the two groups (*p* > 0.05, Figures 4E,I,J). As one of the APP shear products, the numbers of cortical Aβ-positive plaques in APP/PS1 mice were examined via immunohistochemistry. Relative to vehicle treatment, D-Pen-CS/β-GP treatment reduced the number (*p* < 0.05; Figures 4K,L) of Aβ-positive plaques. Plaque size was also smaller, and plaques became more dispersed in mice treated with D-Pen-CS/β-GP (*p* < 0.05; Figures 4K,M). Based on our findings, we speculated that the activation of non-amyloidogenic processing may inhibit the production of Aβ and the accumulation of SPs, which may in turn halt the progression of the enhancement of glial cell activity to some extent. GFAP-positive astrocytes with round nuclei and a ramified form with many fine processes were observed around bright, solid SPs in APP/PS1 mice. D-Pen hydrogel weakened the activation of astrocytes around the SPs, which had become more diffuse, with smaller nuclei and shorter processes (Figure 4N). Western blot analysis also revealed that GFAP protein levels had been attenuated by D-Pen treatment (*p* < 0.05, Figures 4O,P).

### D-Pen-CS/β-GP Treatment Enhanced Non-amyloidogenic Processing *in vitro*

**FIGURE 5:**
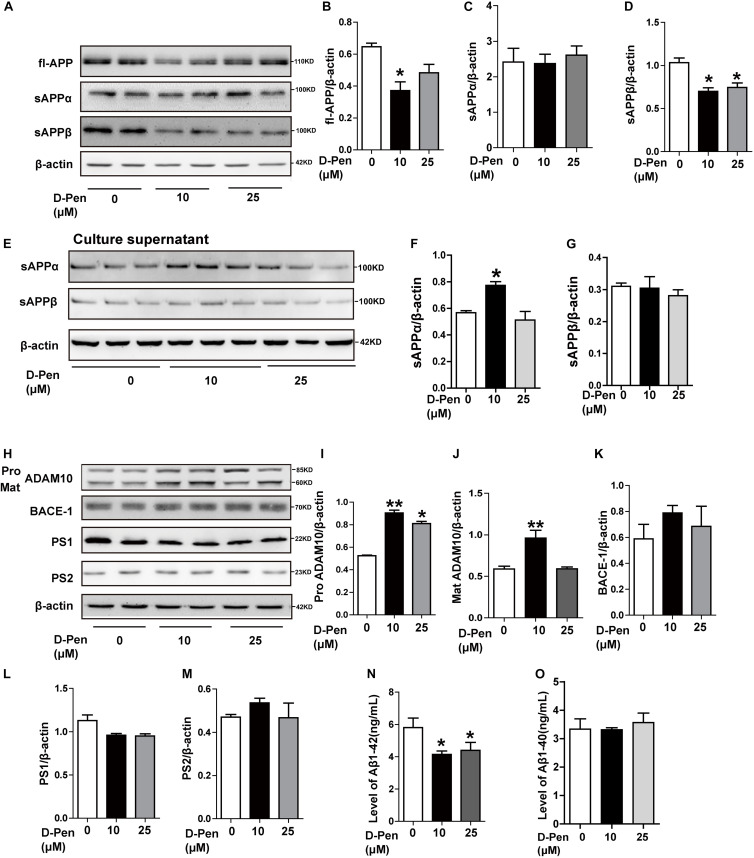
The effect of D-Pen on APP processing in N2a-sw cells. Representative immunoblots and quantification of the expression of APP-processing proteins in N2a-sw cells treated with medium containing 10 and 25 μM D-Pen. **(A–D)** APP-processing proteins including fl-APP, sAPPα, and sAPPβ were detected by Western blot analysis. **(E–G)** sAPPα and sAPPβ protein levels were detected in media by Western blot analysis. **(H–M)** The enzymes involved in ADAM10, BACE1, PS2, and PS1 expression were determined via immunoblot analysis. β-actin was used as an internal control. Data are presented as the mean ± standard error of the mean (SEM) at least three independent experiments by one-way analysis of variance. Extracellular Aβ1-40 and 1-42 secretions **(N,O)** were measured via enzymatic cleavage assays. Statistical significance in multiple comparisons was determined by one-way analysis of variance, *p < 0.05, **p < 0.01 compared to the control group.

Our *in vitro* analyses also indicated that APP processing was affected by D-Pen. In N2a cells overexpressing the APP Sw mutation, Western blot analyses indicated that full-length APP (fl-APP) expression had decreased following the treatment with 10 μM D-Pen (*p* < 0.05, Figures 5A,B), although these changes were not significant at 25 μM (*p* > 0.05; Figures 5A,B). Secreted sAPPα protein levels were increased in media not in cell pellets under 10 μM D-Pen treatment (Figures 5C,E,F). The expression of soluble APP-β (sAPPβ) in cells decreased following the treatment with media containing 10 or 25 μM D-Pen (*p* < 0.05, Figure 5D), while secreted sAPPβ protein levels were not changed in media (*p* > 0.05, Figures 5E,G). The levels of pro-ADAM10 and mature-ADAM10 expression also increased following the treatment with 10 μM D-Pen (*p* < 0.05, Figures 5H,I,J). In contrast, 25 μM D-Pen treatments only increased the protein levels of pro-ADAM10. BACE1 protein levels did not change following the initial treatment with 10 and 25 μM D-Pen (*p* > 0.05; Figures 5H,K) and the same treatments had no significant effect on PS1 and PS2 (*p* > 0.05; Figures 5L,M). ELISA results revealed that Aβ1-42 secretion into the extracellular space had decreased following treatment with D-Pen (Figure 5N), and the secreted Aβ1-40 in the extracellular space had not changed by the treatment with D-Pen (Figure 5O).

**FIGURE 6:**
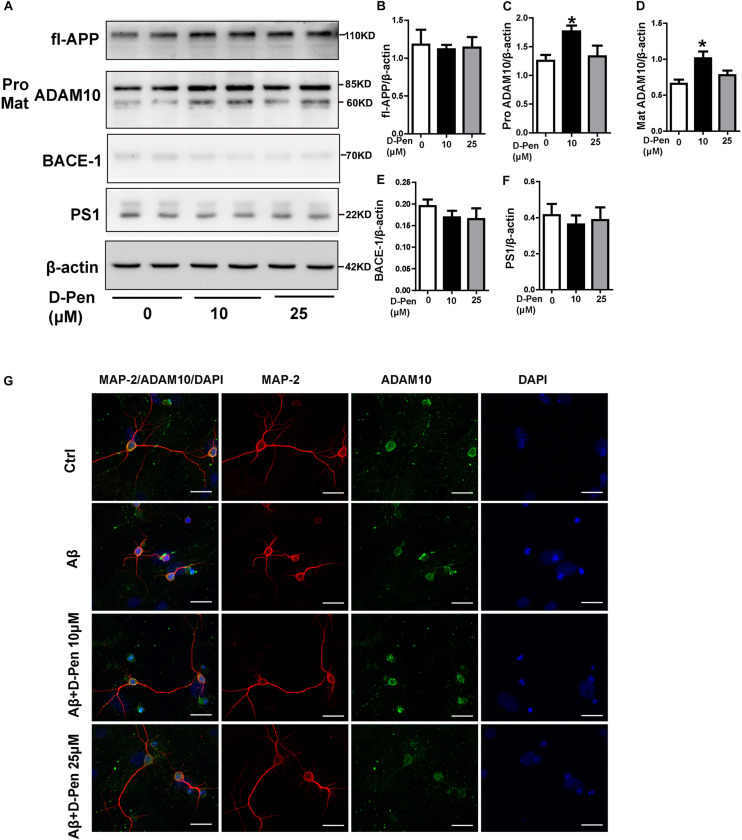
The effect of D-Pen on APP processing in N2a cells and D-Pen enhanced expression of ADAM10 in primary neurons injured by Aβ. **(A)** Representative immunoblots and quantification of the expression of APP and enzymes proteins in N2a cells treated with medium containing 10 and 25 μM D-Pen. **(B)** The protein levels of APP were detected by Western blot analysis. **(C–F)** The enzymes involved in ADAM10, BACE1 and PS1 expression were determined via immunoblot analysis. Data are presented as the mean ± standard error of the mean (SEM) at least three independent experiments. Statistical significance in multiple comparisons was determined by one-way analysis of variance ^∗^*p* < 0.05 compared to the control group. **(G)** Representative immunofluorescence images of ADAM10 and MAP-2 staining in primary cortical neuron (scale bar = 25 μm).

To ensure the effects of D-Pen to ADAM10 expression specifically, we also investigated the expressions of APP and related cleavage enzymes in N2a cells. The result was interesting that there was an expected results with 10 μM D-Pen treatment only increased ADAM10 in N2a cells (Figures 6A–F). Immunofluorescence staining further confirmed that D-Pen could attenuate Aβ oligomer-induced neuronal synaptic damage and ADAM10 expression reduction in primary cortical neurons (Figure 6G). The expressions of mature-ADAM10 reduced by Aβ oligomers were alleviated to different extents by 10 or 25 μM D-Pen treatment, respectively, in primary cortical neurons (Supplementary Figures 3A–G).

Interestingly, the *in vitro* results showed that 10 μM D-Pen efficiently affected the level of ADAM10 and sAPPα secretion. Considering *in vivo* and *in vitro* results, we speculated that the mainly effect of D-Pen might be regulate APP non-amyloid pathway thereby leading to decreases in Aβ production and deposition.

### D-Pen Hydrogel Regulated Non-amyloidogenic Processing via Melatonin Receptor 1 and the PKA/ERK/CREB Pathway

**FIGURE 7:**
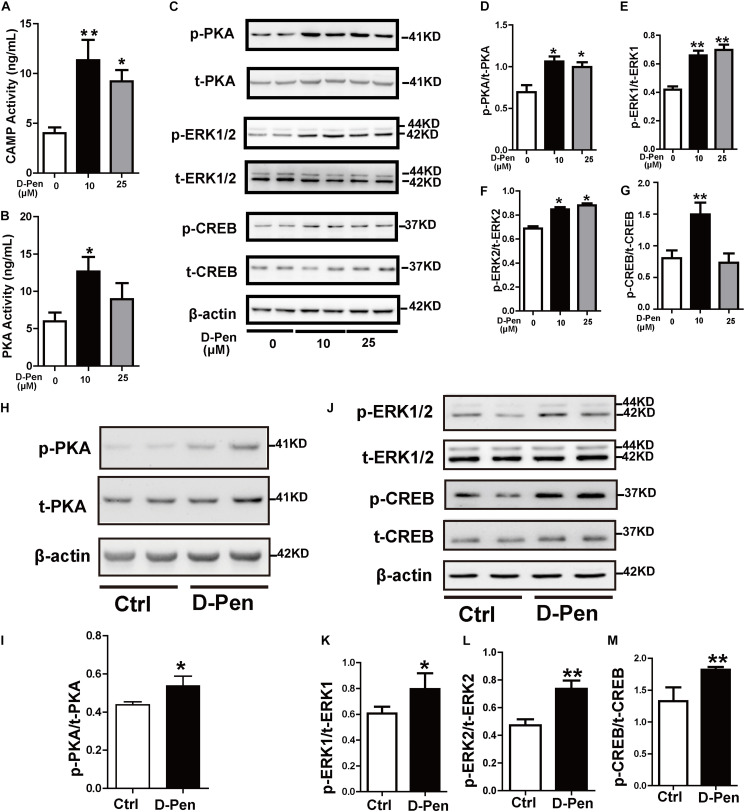
D-Pen upregulated the PKA/ERK/CREB signaling pathway *in vitro* and *in vivo*. The cAMP **(A)** and PKA **(B)** activation in N2a-sw cells was detected via ELISA. Immunoblotting images **(C)** and quantification of p-PKA/PKA **(D)**, p-ERK/ERK **(E–F)**, and p-CREB/CREB **(G)** expression are shown in the figure. β-actin was used as an internal control. N2a-sw cells were treated with 10 or 25 μM of D-Pen in FBS-free DMEM for 24 h. Data are represented as the mean ± standard error of the mean (SEM) at least three independent experiments. **(H,J)** The cerebral cortices of mice from the vehicle control and D-Pen groups were lysed (*n* = 5) and quantitation of the proteins including p-PKA, p-ERK1/2, p-CREB, total-PKA, total-ERK1/2, and total-CREB were estimated by Western blot analysis. Levels of p-PKA **(I)**, p-ERK1/2 **(K,L)**, and p-CREB **(M)** were increased following treatment with D-Pen hydrogel. Statistical significance in multiple comparisons was determined by one-way analysis of variance, *p < 0.05, **p < 0.01 compared to the control group.

Cyclic AMP response element binding protein (CREB) as a nuclear transcription factor stimulates the transcription of the ADAM10 promoter (Shukla et al., 2015). We attempted to verify whether D-Pen promotes ADAM10 expression through CREB activation. Firstly, PKA and cAMP activations were stimulated by D-Pen *in vitro* treatment (Figures 7A,B). Then, Western blot analyses revealed that the protein levels of p-PKA increased after treatment with D-Pen hydrogel (Figures 7C,D,H,I), and the downstream proteins (p-ERK and p-CREB) exhibited different degrees of upregulation *in vivo* and *in vitro* (Figures 7C,E–G,J,K–M). D-Pen treatment also upregulated the expression of the PKA-ERK-CREB pathway in primary neurons injured by Aβ (Supplementary Figures 3H–L). Therefore, we could preliminarily infer that D-Pen regulated the PKA/ERK/CREB signaling pathway according to *in vivo* and *in vitro* experiments.

**FIGURE 8:**
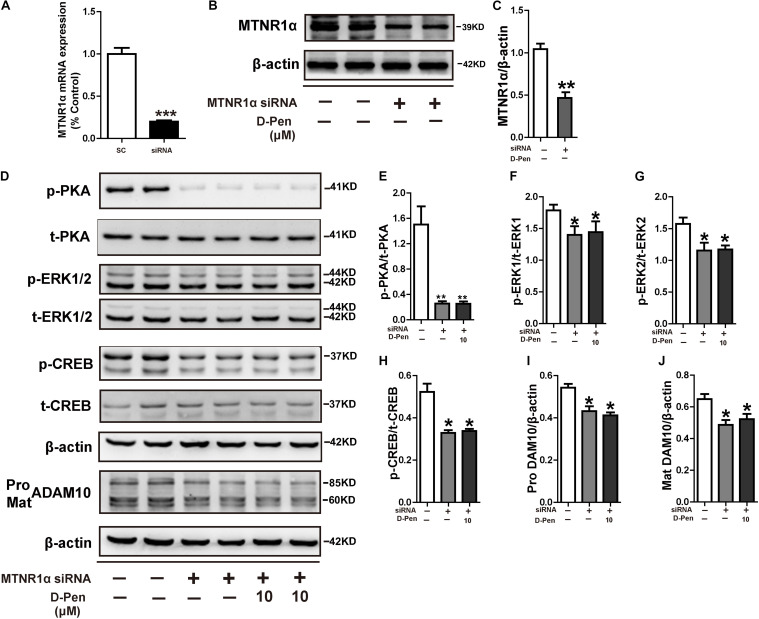
D-Pen regulated the PKA/ERK/CREB signaling pathway through melatonin receptor-1. **(A)** Down-regulation of *MTNR1*α mRNA expression by the recombinant plasmids, respectively. The mRNA expression level was detected by the real-time PCR assay. Immunoblotting images **(B)** and quantification **(C)** showed that MTNR1α was downregulated in N2a-sw cells. Immunoblots **(D)** and quantification results showed p-PKA/PKA **(E)**, p-ERK/ERK **(F,G)**, p-CREB/CREB **(H)** and pro-ADAM10 **(I)** or mature ADAM10 **(J)** in N2a-sw cells with knockdown MTNR1α, following which they were incubated with 10 μM D-Pen. Data are represented as the mean ± standard error of the mean (SEM) at least three independent experiments. Statistical significance in multiple comparisons was determined by one-way analysis of variance, *p < 0.05, **p < 0.01 compared to the control group.

We have demonstrated copper chelators can regulate the MTNR signaling pathway to affect APP processing, therefore, we speculated that D-Pen also induced upregulation of ADAM10 via MTNR-dependent process *in vitro* and *in vivo* (Galano et al., 2015; Shukla et al., 2015). Firstly, Western blot confirmed that the treatment with D-Pen hydrogel did not affect the expression of MTNR1α and MTNR1β in the cerebral cortex and in N2a-sw cells (Supplementary Figure 4). Then, we knocked down the mRNA expressions of *MTNR1*α and *MTNR1*β (Figure 8A and Supplementary Figure 5A) and found that the protein levels of MTNR1α were successfully reduced to approximately 53.84% (Figures 8B,C) and MTNR1β expression was reduced to approximately 50.91% compared with control non-targeting siRNA (Supplementary Figures 5B,C). Western blot results showed that the decrease of MTNR1α expression blocked D-Pen activation of downstream p-PKA, p-ERK, and p-CREB and also inhibited ADAM10 expression (Figures 8D–J). Down-regulation of MTNR1β did not affect the expression of downstream p-PKA, p-ERK, p-CREB, and ADAM10; even D-Pen treatment also failed to activate PKA, ERK, CREB phosphorylation levels and could not increase ADAM10 expression (Supplementary Figures 5D–J).

**FIGURE 9:**
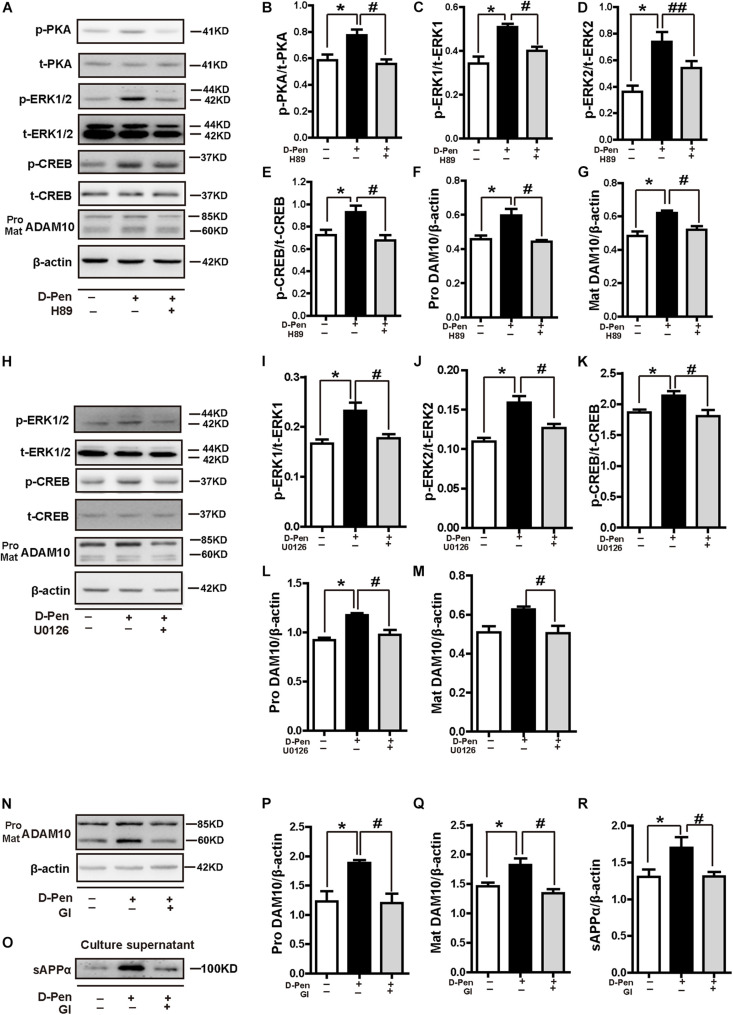
D-penicillamine (D-Pen) regulated the PKA/ERK/CREB signaling pathway to stimulate the expression of ADAM10 and the secretion of sAPPα. **(A)** N2a-sw cells were treated with 10 μM D-Pen or pretreated with H89 (5 μM) for 2 h, following which they were incubated with 10 μM D-Pen in FBS-free DMEM for 24 h. Quantitation of the proteins, including p-PKA/PKA **(B)**, p-ERK1/ERK1 **(C)**, p-ERK2/ERK2 **(D)**, p-CREB/CREB **(E)**, and pro-ADAM10 **(F)** or mature ADAM10 **(G)** were shown. **(H)** N2a-sw cells were treated with 10 μM D-Pen or pretreated with U0126 (10 μM) for 2 h, following which they were incubated with 10 μM D-Pen in FBS-free DMEM for 24 h. Quantitation of the proteins, including p-ERK1/ERK1 **(I)**, p-ERK2/ERK2 **(J)**, p-CREB/CREB **(K)**, and pro-ADAM10 **(L)** or mature ADAM10 **(M)** were analyzed. N2a-sw cells were treated with 10 μM D-Pen or pretreated with GI 254023X (10 μM) for 2 h, following which they were incubated with 10 μM D-Pen in DMEM without FBS for 24 h. Representative images of ADAM10 **(N)** and sAPPα **(O)** were shown by Western blot. Moreover, quantification analysis of the proteins, including pro-ADAM10 **(P)** or mature ADAM10 **(Q)** and secreted sAPPα **(R)** in N2a-sw cells were presented in the figure. At least three independent experiments were executed. Data are represented as the mean ± standard error of the mean (SEM) at least three independent experiments. Statistical significance in multiple comparisons was determined by one-way analysis of variance, *p < 0.05, compared to the control group, ^#^*p* < 0.05, ^##^*p* < 0.01 compared to the D-Pen group.

To further elucidate the possible mechanisms of PKA/ERK/CREB in the regulation of ADAM10 expression, we treated N2a-sw cells with the pharmacological PKA inhibitor H89, ERK1/2 inhibitor U0126 or ADAM10 antagonist GI 254023X in the presence or absence of D-Pen (10 μM). The results indicated that both H89 (Figures 9A–G) and U0126 (Figures 9H–M) inhibited the CREB production and decreased the ADAM10 expression that was induced by D-Pen. At the same time, the effects of GI 254023X inhibition affected the increase of ADAM10 expression (Figures 9N,P,Q) and sAPPα secretion (Figures 9O,R) induced by D-Pen treatment. Consistent with our previous observations following tetrathiomolybdate (TM) or bathocuproine sulfonate (BCS) treatment (Wang et al., 2018), D-Pen stimulated the expression of ADAM10 and the secretion of sAPPα via the MTNR1α-dependent signaling pathway.

## Discussion

In the present study, we focused on the ability of D-Pen to stimulate ADAM10 expression and promote non-amyloidogenic APP processing, which may aid in the treatment of AD. Some studies have shown that the oral D-Pen administration can decrease copper levels in red blood cells and reduce copper-zinc superoxide dismutase activity and that D-Pen nanoparticles can depolymerize copper-Aβ (1–42) aggregates *in vitro* (Squitti et al., 2002; Cui et al., 2005). In these studies, the ability of D-Pen to penetrate the BBB and the bioavailability of D-Pen were not determined, suggesting that further clarifications were required. Given that its D-Pen hydrophilicity restricts the delivery through the cell membrane and BBB, we developed a novel hydrogel that allows D-Pen transport to the brain. Our results clarified that intranasal administration of D-Pen mainly upregulated ADAM10 expression to achieve better therapeutic effects against AD, which was via MTNR1α and stimulated the PKA/ERK/CREB pathway.

Hydrogel delivery systems are highly biocompatible, enable drugs to bypass the BBB due to their similarity to the native extracellular matrix, and exhibited target drug delivery, reduction of the peripheral toxicity and controlled drug release kinetics (Sosnik and Seremeta, 2017; Aderibigbe, 2018). CS could significantly enhance the delivery of larger peptides and proteins as well as the absorption of low molecular weight polar drugs across the nasal epithelial membrane. The main mechanism involves the interaction of CS with the anionic counterparts in the mucous layer of the nasal cavity through its own positively charged amino group, and instantly opening the tight junction of the olfactory and respiratory epithelial cells in the nasal cavity, thus affecting the permeability of the epithelial membrane (Vllasaliu et al., 2010). In the present study, we focused on the development and characterization of a biocompatible, thermo-sensitive CS gel in which D-Pen could be dissolved. Our sensitivity experiments indicated that the D-Pen gel becomes gelatinous at 37°C. At low temperatures, the glycerol molecules of β-GP form a water barrier surrounding the CS molecules to maintain the native form of CS chains and inhibit their aggregation (Cho et al., 2005). As the temperature increases, the strengthening of CS-CS interactions results in a phase transition from liquid to gel. Thus, the chemical actions of the D-Pen/CS gel do not change, but D-Pen utilization is improved. The high performance liquid chromatography (HPLC) results confirmed that D-Pen delivered from the hydrogel could enter into the brain; however, its targeting was not clear.

As metal ions participate in essential neural functions, removing them with high affinity chelating agents might be problematic (Faux et al., 2010; Wang et al., 2012). However, it is more appropriate to describe AD as a turbation of metal dyshomeostasis, rather than as a simple metal-overload disease. Thus, it is more important to reduce the local aggregation of metal ions and maintain metal homeostasis. Although Mumper et al. demonstrated the ability of D-Pen nanoparticles to degrade copper-Aβ *in vitro*, they did not explore its release or chelating effects *in vivo* (Cui et al., 2005). In the present study, the ICP-MS results showed that the contents of iron, copper and zinc in the cerebral cortex of APP/PS1 mice was decreased after D-Pen treatment. *In vitro* experiments also confirmed that D-Pen reduced intracellular metal content was not associated with cytotoxic effects, indicating that its chelating effects did not affect the normal physiological cell function. D-pen as a metal chelators could capture and bind metal ions which can deplete the total pool of bioavailable metals extracellularly or compete with endogenous ligand (Duce and Bush, 2010). In the present study, we aimed to determine the mechanism by which D-Pen could to halt the progression of AD by activating key enzymes in its non-amyloidogenic pathways. The generation and deposition of Aβ is directly related to the relative proteolytic efficiency of APP in amyloidogenic and non-amyloidogenic pathways. Both *in vivo* and *in vitro* experiments confirmed that D-Pen did reduce the number of Aβ-positive plaques and the level of Aβ secretion. In addition to D-Pen chelating metals in senile plaques to reduce the accumulation of Aβ, our *in vitro* and *in vivo* results revealed that ADAM10 significantly increased the level of APPα expression in the brain and extracellular space. We insured that D-Pen mainly regulated the expression of ADAM10 and promoted the expression of sAPPα, thereby attenuating Aβ production. During APP cleavage, ADAM10 plays an important role in reducing Aβ generation (Peron et al., 2018). The sAPPα cleavage fragment generated by ADAM10 can largely restore the behavioral and electrophysiological abnormalities of APP-deficient mice (Ring et al., 2007) and rescue the hippocampal neuronal damage induced by neurotoxicity, glucose deprivation, and Aβ toxicity (Furukawa et al., 1996; Barger and Harmon, 1997). If only using a sw-APP model, there was going to be a preferential processing of APP down the amyloidogenic pathway due to the mutation, we demonstrated a similar outcome was observed with endogenously expressed APP in N2a and primary neurons. Therefore, we could speculate that ADAM10 expression was particularly adjusted by D-Pen. Moreover, our results showed that D-Pen could not only increase the expressions of ADAM10 in cells and tissues, but the effects on the expressions of BACE-1 were inconsistent in tissues and cells, which may be due to the activation of non-amyloid pathways. The resulting changes in β-secretases could also be related to the chelation of D-Pen (Stelmashook et al., 2014; Gerber et al., 2017), which requires further research.

Consequently, we investigated the molecular mechanisms by which D-Pen activates ADAM10 expression and stimulates the non-amyloidogenic α-secretase pathway. Previous investigations have reported that melatonin acts via the MTNRs to induce G-protein/PKC/ERK activation and elevate ADAM10 levels in HEK293 and neuronal SH-SY5Y cells, and that α-secretase activity is fully dependent on melatonin receptors (Panmanee et al., 2015; Shukla et al., 2015). In our previous study, we observed that copper chelators induced ADAM10 production, a process controlled by MT_1__/__2_/CREB-dependent signaling pathways (Wang et al., 2018). In this context, after knocking down *MTNR1*α, D-Pen blocked the activations of PKA, ERK and CREB of the MT downstream and did not influence the expression of ADAM10. After knocking down *MTNR1*β, D-Pen had no effect on the expressions of PKA, ERK, CREB, and ADAM10. The above results indicated that D-Pen selectively regulated downstream signaling pathways through MTNR1α but not through MTNR1β. We observed that D-Pen attenuated the phosphorylation of ERK and CREB, as well as the expression of ADAM10, after blockade by H89 and U0126. Because D-Pen can promote cAMP through G-coupled receptors, we should also consider the indirect effect of cAMP/PKA on CREB. This finding can be explained by the fact that activation of the ERK pathway is known to induce Ser133 residue phosphorylation of CREB and subsequent CREB-dependent gene expression (Kwok et al., 1994; Mei et al., 2006). Combined with the fact that ERK and CREB phosphorylation levels and ADAM10 expression significantly increased by D-Pen treatment alone *in vitro* and *in vivo*, we conclude that D-Pen regulated ADAM10 via the PKA/ERK/CREB signaling pathway. Additionally, it cannot be ignored that the D-Pen’s direct function as a metal chelator could competitively compound with zinc, iron and copper to redistribute the metal ions in the brain. Although it is not possible to dismiss the possibility that D-Pen may also act through some additional inducible factors, we can reasonably draw a conclusion based on our experimental results that D-Pen upregulated the transcription and expression of ADAM10 mainly through the activation of CREB. Thus, our results firmly establish that D-Pen treatment upregulated ADAM10 activity by stimulating the MTNR1α/PKA/ERK/CREB signaling pathway. On the other hand, we found that 25 μM D-Pen had little effect on the downstream signaling pathways. The maintenance of cell homeostasis may be a key factor in determining the related proteins of the signaling pathway (Sun et al., 2010; Galano et al., 2015; Boyd et al., 2020). Although the MTT results did not show the effect of D-Pen on the cells survival, the activity of LDH increased and the activity of SOD decreased in the 25 μM D-Pen medium (data not shown). These results indicated that 25 μM D-Pen had an impact on the homeostasis of the intracellular environment, and no obvious effect on the expressions of the downstream proteins. Althrough, more data, such as the changes in the apoptosis-related factors and autophagy signaling pathways etc, are required in future research.

In accordance with these findings, D-Pen improved the learning and memory abilities in the AD model employed in the present study. The production of a neuroprotective sAPPα fragment attenuated the learning deficit of the double-transgenic ADAM10 × APP_[V__717__*I]*_ mice (Postina et al., 2004). However, we could not determine whether Aβ reduction or sAPPα production plays the key role based on behavioral tests and cell survival experiments. There are still some aspects of our research that need to be supplemented. For example, it has proven that D-Pen could reduce the contents of metal ions in the brain, but its therapy on metal dyshomeostasis related effects needs to be further improved exploration. Although we established a method of nasal gel administration that improves the bioavailability of D-Pen, the release profiles of D-Pen in the nervous system and drug toxicology require further validation.

## Conclusion

Our findings showed that D-Pen-CS/β-GP hydrogel treatment activated the ADAM10 via MTNR1α pathway, which significantly relieved Aβ burden and ameliorated behavioral deficits in APP/PS1 mice. These results highlight the role of ADAM10 as a key therapeutic target for D-Pen, indicating that the D-Pen may be an effective drug for the targeted treatment of AD pathology.

## Data Availability Statement

The raw data supporting the conclusions of this article will be made available by the authors, without undue reservation.

## Ethics Statement

The animal study was reviewed and approved by Animal Ethics Committee of the China Medical University.

## Author Contributions

MZ mainly researched the data. HK, PZ, WZ, and HX researched the data. XZ, WL, and TW generated and validated the mouse model. FG contributed the experimental method guidance. CG contributed to discussion. ZW reviewed and edited the manuscript. HG designed and wrote the manuscript. HG was the guarantor of this work and, as such, had full access to all the data in the study and takes responsibility for the integrity of the data and the accuracy of the data analysis. All authors contributed to the article and approved the submitted version.

## Conflict of Interest

The authors declare that the research was conducted in the absence of any commercial or financial relationships that could be construed as a potential conflict of interest.

## References

[B1] Aderibigbe B. A. (2018). In situ-based gels for nose to brain delivery for the treatment of neurological diseases. *Pharmaceutics* 10:40. 10.3390/pharmaceutics10020040 PMC6027251 29601486

[B2] Agrawal M. Saraf S. Antimisiaris S. G. Chougule M. B. Shoyele S. A. Alexander A. (2018). Nose-to-brain drug delivery: an update on clinical challenges and progress towards approval of anti-Alzheimer drugs. *J. Control Release* 281 139–177. 10.1016/j.jconrel.2018.05.011 29772289

[B3] Alexander A. Saraf S. (2018). Nose-to-brain drug delivery approach: a key to easily accessing the brain for the treatment of Alzheimer’s disease. *Neural Regen. Res.* 13 2102–2104. 10.4103/1673-5374.241458 30323136 PMC6199953

[B4] Atrian-Blasco E. Conte-Daban A. Hureau C. (2017). Mutual interference of Cu and Zn ions in Alzheimer’s disease: perspectives at the molecular level. *Dalton Trans.* 46 12750–12759. 10.1039/c7dt01344b 28937157 PMC5656098

[B5] Barger S. W. Harmon A. D. (1997). Microglial activation by Alzheimer amyloid precursor protein and modulation by apolipoprotein E. *Nature* 388 878–881. 10.1038/42257 9278049

[B6] Boyd S. D. Ullrich M. S. Skopp A. Winkler D. D. (2020). Copper sources for Sod1 activation. *Antioxidants (Basel)* 9:500. 10.3390/antiox9060500 32517371 PMC7346115

[B7] Bulloj A. Leal M. C. Xu H. Castano E. M. Morelli L. (2010). Insulin-degrading enzyme sorting in exosomes: a secretory pathway for a key brain amyloid-beta degrading protease. *J. Alzheimers Dis.* 19 79–95. 10.3233/JAD-2010-1206 20061628 PMC4414343

[B8] Bush A. I. (2003). Copper, zinc, and the metallobiology of Alzheimer disease. *Alzheimer Dis. Assoc. Disord.* 17 147–150. 14512827 10.1097/00002093-200307000-00005

[B9] Bush A. I. Tanzi R. E. (2008). Therapeutics for Alzheimer’s disease based on the metal hypothesis. *Neurotherapeutics* 5 421–432. 10.1016/j.nurt.2008.05.001 18625454 PMC2518205

[B10] Casettari L. Illum L. (2014). Chitosan in nasal delivery systems for therapeutic drugs. *J. Control Release* 190 189–200. 10.1016/j.jconrel.2014.05.003 24818769

[B11] Cho J. Heuzey M. C. Begin A. Carreau P. J. (2005). Physical gelation of chitosan in the presence of beta-glycerophosphate: the effect of temperature. *Biomacromolecules* 6 3267–3275. 10.1021/bm050313s 16283755

[B12] Cui Z. Lockman P. R. Atwood C. S. Hsu C. H. Gupte A. Allen D. D. (2005). Novel D-penicillamine carrying nanoparticles for metal chelation therapy in Alzheimer’s and other CNS diseases. *Eur. J. Pharm. Biopharm.* 59 263–272. 10.1016/j.ejpb.2004.07.009 15661498

[B13] Delangle P. Mintz E. (2012). Chelation therapy in Wilson’s disease: from D-penicillamine to the design of selective bioinspired intracellular Cu(I) chelators. *Dalton Trans.* 41 6359–6370. 10.1039/c2dt12188c 22327203

[B14] Duce J. A. Bush A. I. (2010). Biological metals and Alzheimer’s disease: implications for therapeutics and diagnostics. *Prog. Neurobiol.* 92 1–18. 10.1016/j.pneurobio.2010.04.003 20444428

[B15] Durairajan S. S. Liu L. F. Lu J. H. Koo I. Maruyama K. Chung S. K. (2011). Stimulation of non-amyloidogenic processing of amyloid-beta protein precursor by cryptotanshinone involves activation and translocation of ADAM10 and PKC-alpha. *J. Alzheimers Dis.* 25 245–262. 10.3233/JAD-2011-102085 21403388

[B16] Ejaz H. W. Wang W. Lang M. (2020). Copper toxicity links to pathogenesis of Alzheimer’s disease and therapeutics approaches. *Int. J. Mol. Sci.* 21:7660. 10.3390/ijms21207660 33081348 PMC7589751

[B17] Faux N. G. Ritchie C. W. Gunn A. Rembach A. Tsatsanis A. Bedo J. (2010). PBT2 rapidly improves cognition in Alzheimer’s disease: additional phase II analyses. *J. Alzheimers Dis.* 20 509–516. 10.3233/JAD-2010-1390 20164561

[B18] Frederickson C. J. Rampy B. A. Reamy-Rampy S. Howell G. A. (1992). Distribution of histochemically reactive zinc in the forebrain of the rat. *J. Chem. Neuroanat.* 5 521–530. 1476668 10.1016/0891-0618(92)90007-d

[B19] Furukawa K. Sopher B. L. Rydel R. E. Begley J. G. Pham D. G. Martin G. M. (1996). Increased activity-regulating and neuroprotective efficacy of alpha-secretase-derived secreted amyloid precursor protein conferred by a C-terminal heparin-binding domain. *J. Neurochem.* 67 1882–1896. 8863493 10.1046/j.1471-4159.1996.67051882.x

[B20] Galano A. Medina M. E. Tan D. X. Reiter R. J. (2015). Melatonin and its metabolites as copper chelating agents and their role in inhibiting oxidative stress: a physicochemical analysis. *J. Pineal Res.* 58 107–116. 10.1111/jpi.12196 25424557

[B21] Gerber H. Wu F. Dimitrov M. Garcia Osuna G. M. Fraering P. C. (2017). Zinc and copper differentially modulate amyloid precursor protein processing by gamma-secretase and amyloid-beta peptide production. *J. Biol. Chem.* 292 3751–3767. 10.1074/jbc.M116.754101 28096459 PMC5339758

[B22] Guo C. Zhang Y. X. Wang T. Zhong M. L. Yang Z. H. Hao L. J. (2015). Intranasal deferoxamine attenuates synapse loss via up-regulating the P38/HIF-1alpha pathway on the brain of APP/PS1 transgenic mice. *Front. Aging Neurosci.* 7:104. 10.3389/fnagi.2015.00104 26082716 PMC4451419

[B23] Hardy J. A. Higgins G. A. (1992). Alzheimer’s disease: the amyloid cascade hypothesis. *Science* 256 184–185. 1566067 10.1126/science.1566067

[B24] Huang X. Chen Y. Li W. B. Cohen S. N. Liao F. F. Li L. (2010). The Rps23rg gene family originated through retroposition of the ribosomal protein s23 mRNA and encodes proteins that decrease Alzheimer’s beta-amyloid level and tau phosphorylation. *Hum. Mol. Genet.* 19 3835–3843. 10.1093/hmg/ddq302 20650958 PMC2935860

[B25] Illum L. (2000). Transport of drugs from the nasal cavity to the central nervous system. *Eur. J. Pharm. Sci.* 11 1–18. 10913748 10.1016/s0928-0987(00)00087-7

[B26] Kwok R. P. Lundblad J. R. Chrivia J. C. Richards J. P. Bachinger H. P. Brennan R. G. (1994). Nuclear protein CBP is a coactivator for the transcription factor CREB. *Nature* 370 223–226. 10.1038/370223a0 7913207

[B27] Lannfelt L. Blennow K. Zetterberg H. Batsman S. Ames D. Harrison J. (2008). Safety, efficacy, and biomarker findings of PBT2 in targeting Abeta as a modifying therapy for Alzheimer’s disease: a phase IIa, double-blind, randomised, placebo-controlled trial. *Lancet Neurol.* 7 779–786. 10.1016/S1474-4422(08)70167-4 18672400

[B28] Liu B. Moloney A. Meehan S. Morris K. Thomas S. E. Serpell L. C. (2011). Iron promotes the toxicity of amyloid beta peptide by impeding its ordered aggregation. *J. Biol. Chem.* 286 4248–4256. 10.1074/jbc.M110.158980 21147772 PMC3039358

[B29] Liu J. L. Fan Y. G. Yang Z. S. Wang Z. Y. Guo C. (2018). Iron and Alzheimer’s disease: from pathogenesis to therapeutic implications. *Front. Neurosci.* 12:632. 10.3389/fnins.2018.00632 30250423 PMC6139360

[B30] Liu Y. Nguyen M. Robert A. Meunier B. (2019). Metal ions in Alzheimer’s disease: a key role or not? *Acc. Chem. Res.* 52 2026–2035. 10.1021/acs.accounts.9b00248 31274278

[B31] Mei M. Su B. Harrison K. Chao M. Siedlak S. L. Previll L. A. (2006). Distribution, levels and phosphorylation of Raf-1 in Alzheimer’s disease. *J. Neurochem.* 99 1377–1388. 10.1111/j.1471-4159.2006.04174.x 17064357

[B32] Nunez J. (2008). Primary culture of hippocampal neurons from p0 newborn rats. *J. Vis. Exp. JoVE* 895. 10.3791/895 19066540 PMC2872976

[B33] Panmanee J. Nopparat C. Chavanich N. Shukla M. Mukda S. Song W. (2015). Melatonin regulates the transcription of betaAPP-cleaving secretases mediated through melatonin receptors in human neuroblastoma SH-SY5Y cells. *J. Pineal Res.* 59 308–320. 10.1111/jpi.12260 26123100

[B34] Patel R. Aschner M. (2021). Commonalities between copper neurotoxicity and Alzheimer’s disease. *Toxics* 9:4. 10.3390/toxics9010004 33430181 PMC7825595

[B35] Peron R. Vatanabe I. P. Manzine P. R. Camins A. Cominetti M. R. (2018). Alpha-secretase ADAM10 regulation: insights into Alzheimer’s disease treatment. *Pharmaceuticals (Basel)* 11:12. 10.3390/ph11010012 29382156 PMC5874708

[B36] Postina R. Schroeder A. Dewachter I. Bohl J. Schmitt U. Kojro E. (2004). A disintegrin-metalloproteinase prevents amyloid plaque formation and hippocampal defects in an Alzheimer disease mouse model. *J. Clin. Invest.* 113 1456–1464. 10.1172/JCI20864 15146243 PMC406531

[B37] Ring S. Weyer S. W. Kilian S. B. Waldron E. Pietrzik C. U. Filippov M. A. (2007). The secreted beta-amyloid precursor protein ectodomain APPs alpha is sufficient to rescue the anatomical, behavioral, and electrophysiological abnormalities of APP-deficient mice. *J. Neurosci.* 27 7817–7826. 10.1523/JNEUROSCI.1026-07.2007 17634375 PMC6672885

[B38] Ritchie C. W. Bush A. I. Mackinnon A. Macfarlane S. Mastwyk M. MacGregor L. (2003). Metal-protein attenuation with iodochlorhydroxyquin (clioquinol) targeting Abeta amyloid deposition and toxicity in Alzheimer disease: a pilot phase 2 clinical trial. *Arch. Neurol.* 60 1685–1691. 10.1001/archneur.60.12.1685 14676042

[B39] Roberts B. R. Ryan T. M. Bush A. I. Masters C. L. Duce J. A. (2012). The role of metallobiology and amyloid-beta peptides in Alzheimer’s disease. *J. Neurochem.* 120(Suppl. 1) 149–166. 10.1111/j.1471-4159.2011.07500.x 22121980

[B40] Sarvaiya J. Agrawal Y. K. (2015). Chitosan as a suitable nanocarrier material for anti-Alzheimer drug delivery. *Int. J. Biol. Macromol.* 72 454–465. 10.1016/j.ijbiomac.2014.08.052 25199867

[B41] Selkoe D. J. (1991). The molecular pathology of Alzheimer’s disease. *Neuron* 6 487–498. 1673054 10.1016/0896-6273(91)90052-2

[B42] Shukla M. Htoo H. H. Wintachai P. Hernandez J. F. Dubois C. Postina R. (2015). Melatonin stimulates the nonamyloidogenic processing of betaAPP through the positive transcriptional regulation of ADAM10 and ADAM17. *J. Pineal Res.* 58 151–165. 10.1111/jpi.12200 25491598

[B43] Singh V. Xu L. Boyko S. Surewicz K. Surewicz W. K. (2020). Zinc promotes liquid-liquid phase separation of tau protein. *J. Biol. Chem.* 295 5850–5856. 10.1074/jbc.AC120.013166 32229582 PMC7196643

[B44] Sosnik A. Seremeta K. P. (2017). Polymeric hydrogels as technology platform for drug delivery applications. *Gels* 3:25. 10.3390/gels3030025 30920522 PMC6318675

[B45] Spotorno N. Acosta-Cabronero J. Stomrud E. Lampinen B. Strandberg O. T. van Westen D. (2020). Relationship between cortical iron and tau aggregation in Alzheimer’s disease. *Brain* 143 1341–1349. 10.1093/brain/awaa089 32330946 PMC7241946

[B46] Squitti R. Ghidoni R. Simonelli I. Ivanova I. D. Colabufo N. A. Zuin M. (2018). Copper dyshomeostasis in Wilson disease and Alzheimer’s disease as shown by serum and urine copper indicators. *J. Trace Elem. Med. Biol.* 45 181–188. 10.1016/j.jtemb.2017.11.005 29173477

[B47] Squitti R. Rossini P. M. Cassetta E. Moffa F. Pasqualetti P. Cortesi M. (2002). d-penicillamine reduces serum oxidative stress in Alzheimer’s disease patients. *Eur. J. Clin. Invest.* 32 51–59. 11851727 10.1046/j.1365-2362.2002.00933.x

[B48] Stelmashook E. V. Isaev N. K. Genrikhs E. E. Amelkina G. A. Khaspekov L. G. Skrebitsky V. G. (2014). Role of zinc and copper ions in the pathogenetic mechanisms of Alzheimer’s and Parkinson’s diseases. *Biochemistry (Mosc)* 79 391–396. 10.1134/S0006297914050022 24954589

[B49] Stine W. B. Jungbauer L. Yu C. LaDu M. J. (2011). Preparing synthetic Abeta in different aggregation states. *Methods Mol. Biol.* 670 13–32. 10.1007/978-1-60761-744-0_2 20967580 PMC3752843

[B50] Sun Z. W. Zhang L. Zhu S. J. Chen W. C. Mei B. (2010). Excitotoxicity effects of glutamate on human neuroblastoma SH-SY5Y cells via oxidative damage. *Neurosci. Bull.* 26 8–16. 10.1007/s12264-010-0813-7 20101268 PMC5560379

[B51] Thinakaran G. Teplow D. B. Siman R. Greenberg B. Sisodia S. S. (1996). Metabolism of the “Swedish” amyloid precursor protein variant in neuro2a (N2a) cells. Evidence that cleavage at the “beta-secretase” site occurs in the golgi apparatus. *J. Biol. Chem.* 271 9390–9397. 10.1074/jbc.271.16.9390 8621605

[B52] Varea E. Ponsoda X. Molowny A. Danscher G. Lopez-Garcia C. (2001). Imaging synaptic zinc release in living nervous tissue. *J. Neurosci. Methods* 110 57–63. 11564525 10.1016/s0165-0270(01)00417-4

[B53] Vllasaliu D. Exposito-Harris R. Heras A. Casettari L. Garnett M. Illum L. (2010). Tight junction modulation by chitosan nanoparticles: comparison with chitosan solution. *Int. J. Pharm.* 400 183–193. 20727955 10.1016/j.ijpharm.2010.08.020

[B54] Wang T. Wang C. Y. Shan Z. Y. Teng W. P. Wang Z. Y. (2012). Clioquinol reduces zinc accumulation in neuritic plaques and inhibits the amyloidogenic pathway in AbetaPP/PS1 transgenic mouse brain. *J. Alzheimers Dis.* 29 549–559. 10.3233/JAD-2011-111874 22269164

[B55] Wang Z. Zhang Y. H. Zhang W. Gao H. L. Zhong M. L. Huang T. T. (2018). Copper chelators promote nonamyloidogenic processing of AbetaPP via MT1/2/CREB-dependent signaling pathways in AbetaPP/PS1 transgenic mice. *J. Pineal Res.* 65:e12502. 10.1111/jpi.12502 29710396

[B56] West M. J. (1999). Stereological methods for estimating the total number of neurons and synapses: issues of precision and bias. *Trends Neurosci.* 22 51–61. 10092043 10.1016/s0166-2236(98)01362-9

[B57] Yang G. J. Liu H. Ma D. L. Leung C. H. (2019). Rebalancing metal dyshomeostasis for Alzheimer’s disease therapy. *J. Biol. Inorg. Chem.* 24 1159–1170. 10.1007/s00775-019-01712-y 31486954

[B58] Zhang X. Zhong M. Zhao P. Zhang X. Li Y. Wang X. (2019). Screening a specific Zn(ii)-binding peptide for improving the cognitive decline of Alzheimer’s disease in APP/PS1 transgenic mice by inhibiting Zn(2+)-mediated amyloid protein aggregation and neurotoxicity. *Biomater. Sci.* 7 5197–5210. 10.1039/c9bm00676a 31588929

[B59] Zhang Y. W. Liu S. Zhang X. Li W. B. Chen Y. Huang X. (2009). A functional mouse retroposed gene Rps23r1 reduces Alzheimer’s beta-amyloid levels and tau phosphorylation. *Neuron* 64 328–340. 10.1016/j.neuron.2009.08.036 19914182 PMC3846276

[B60] Zhang Z. Miah M. Culbreth M. Aschner M. (2016). Autophagy in neurodegenerative diseases and metal neurotoxicity. *Neurochem. Res.* 41 409–422. 10.1007/s11064-016-1844-x 26869037

