# Supplementary figures and images for: Nasal Delivery of D-Penicillamine Hydrogel Upregulates a Disintegrin and Metalloprotease 10 Expression via Melatonin Receptor 1 in Alzheimer’s Disease Models
Source: Front Aging Neurosci. 2021 Apr 15;13:660249. doi: 10.3389/fnagi.2021.660249 (PMC8081912; doi:10.3389/fnagi.2021.660249)

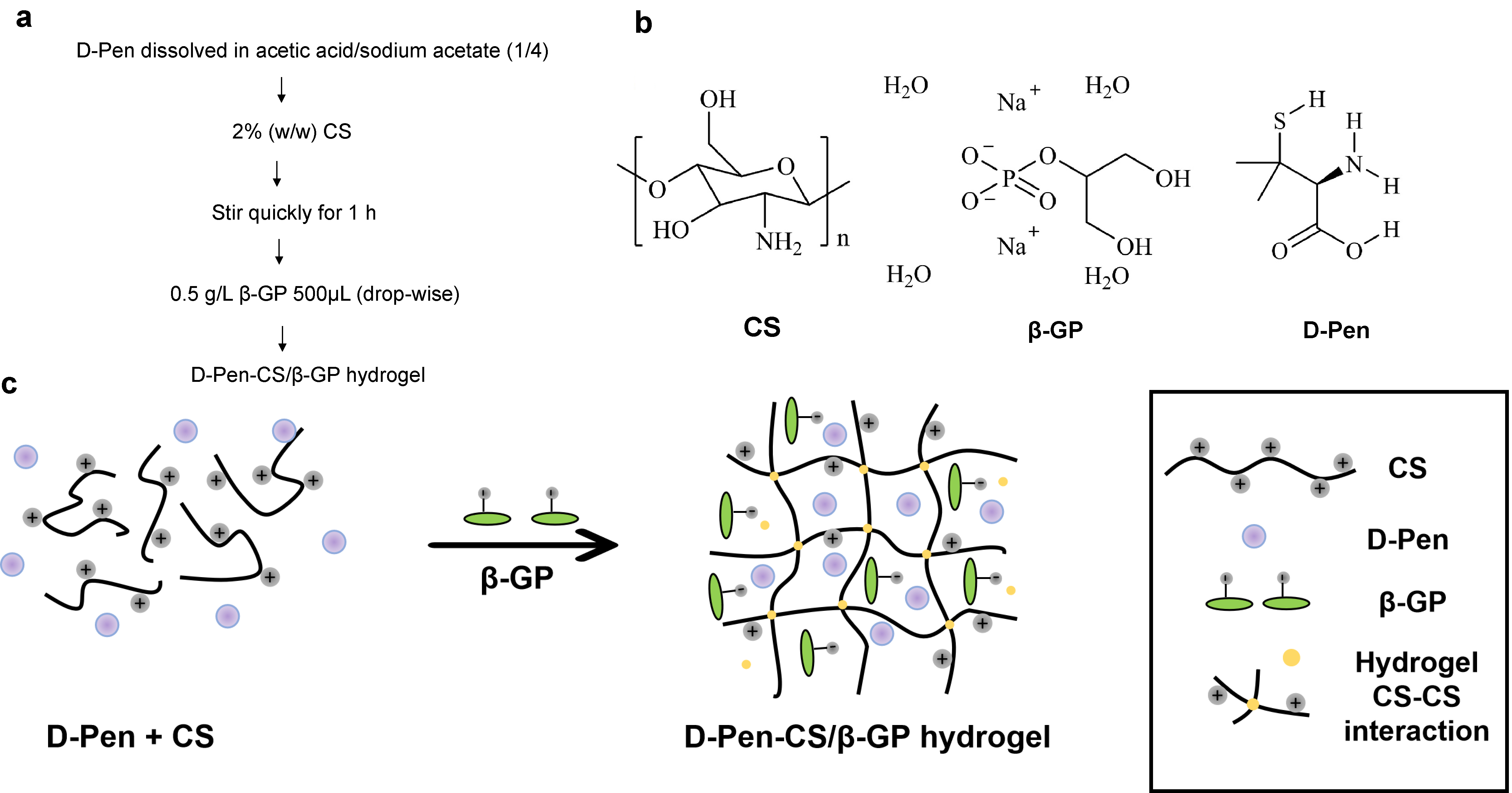

Supplement: Supplementary file 1 [file Image_1.JPEG]

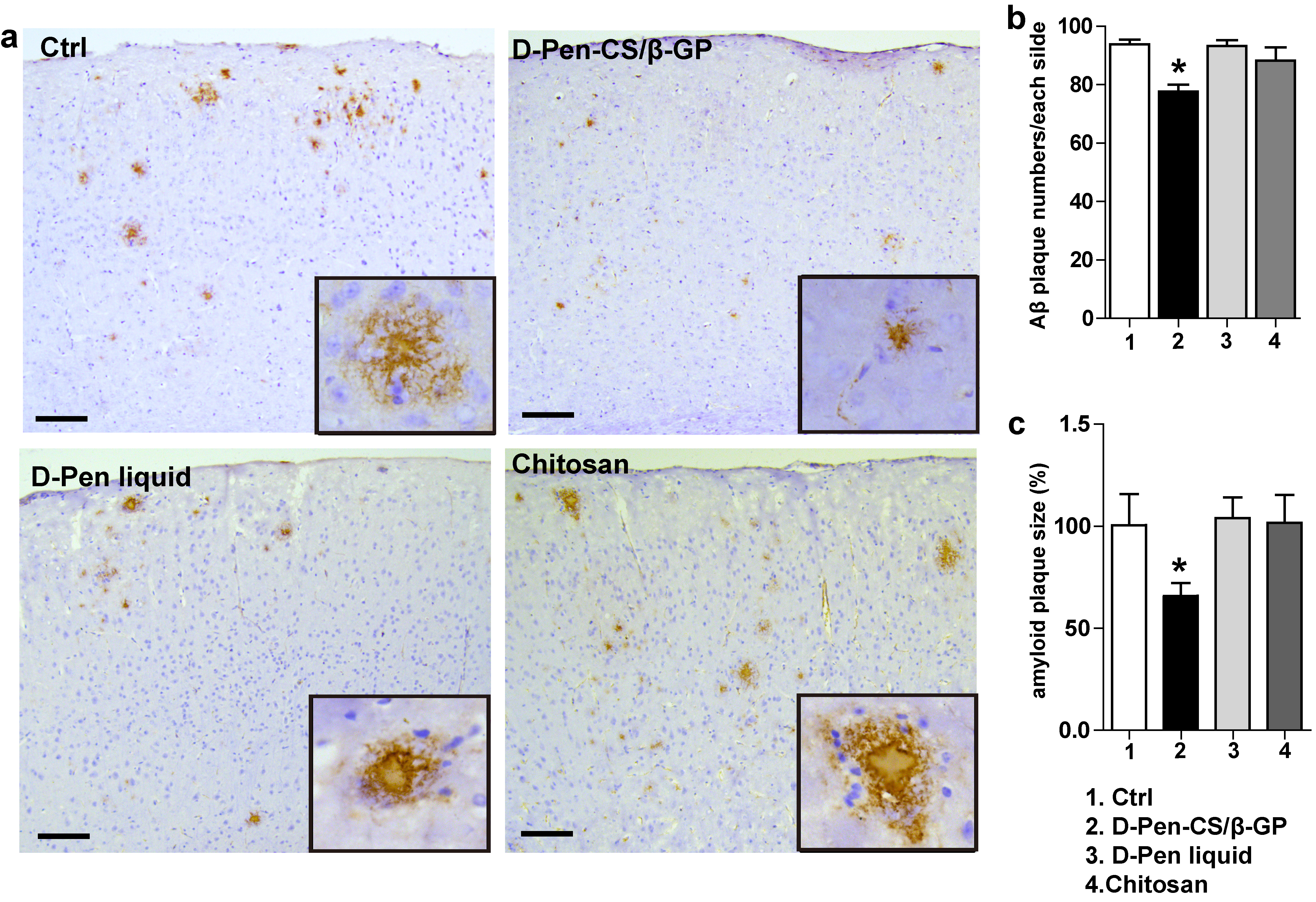

Supplement: Supplementary file 2 [file Image_2.JPEG]

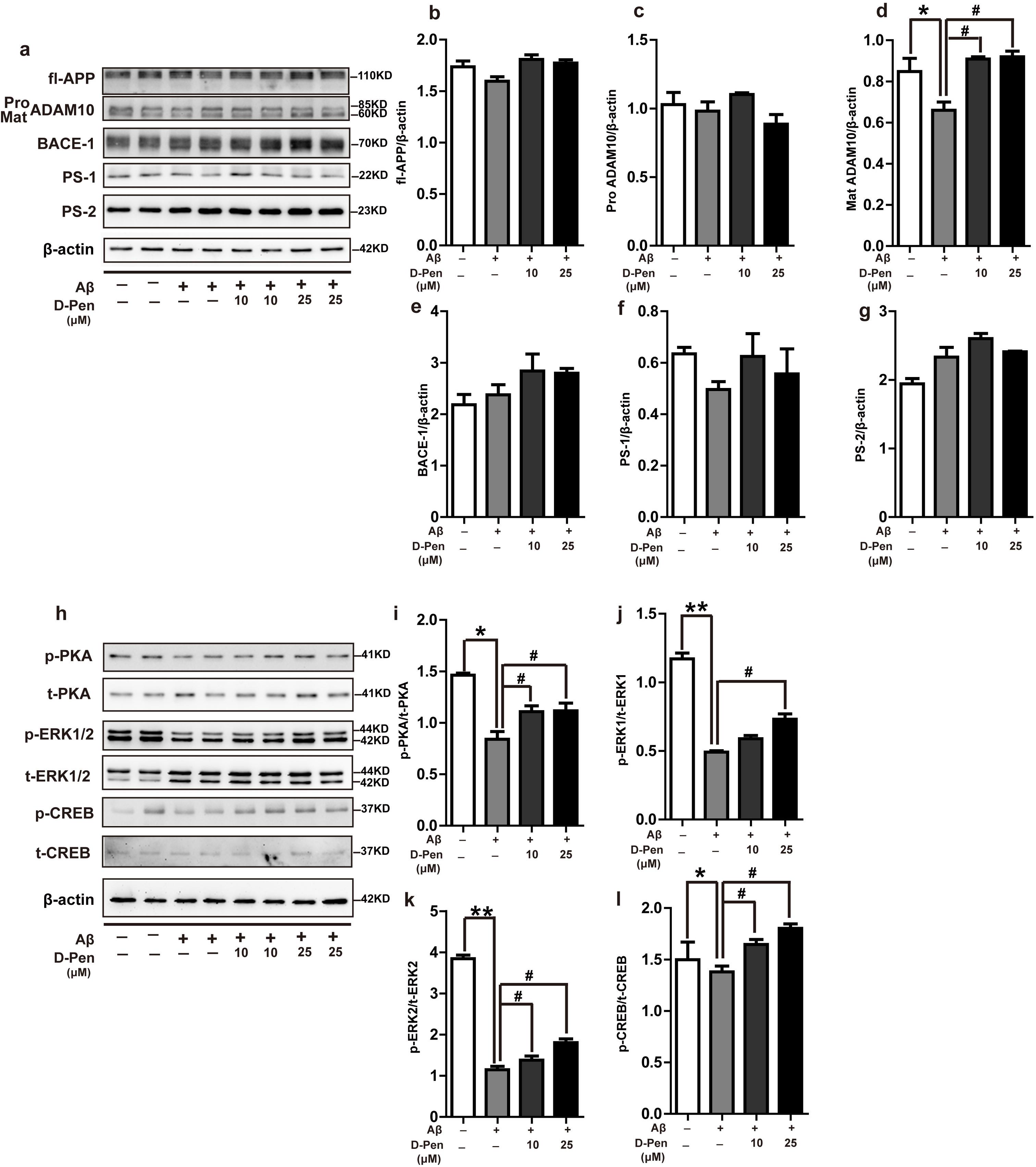

Supplement: Supplementary file 3 [file Image_3.JPEG]

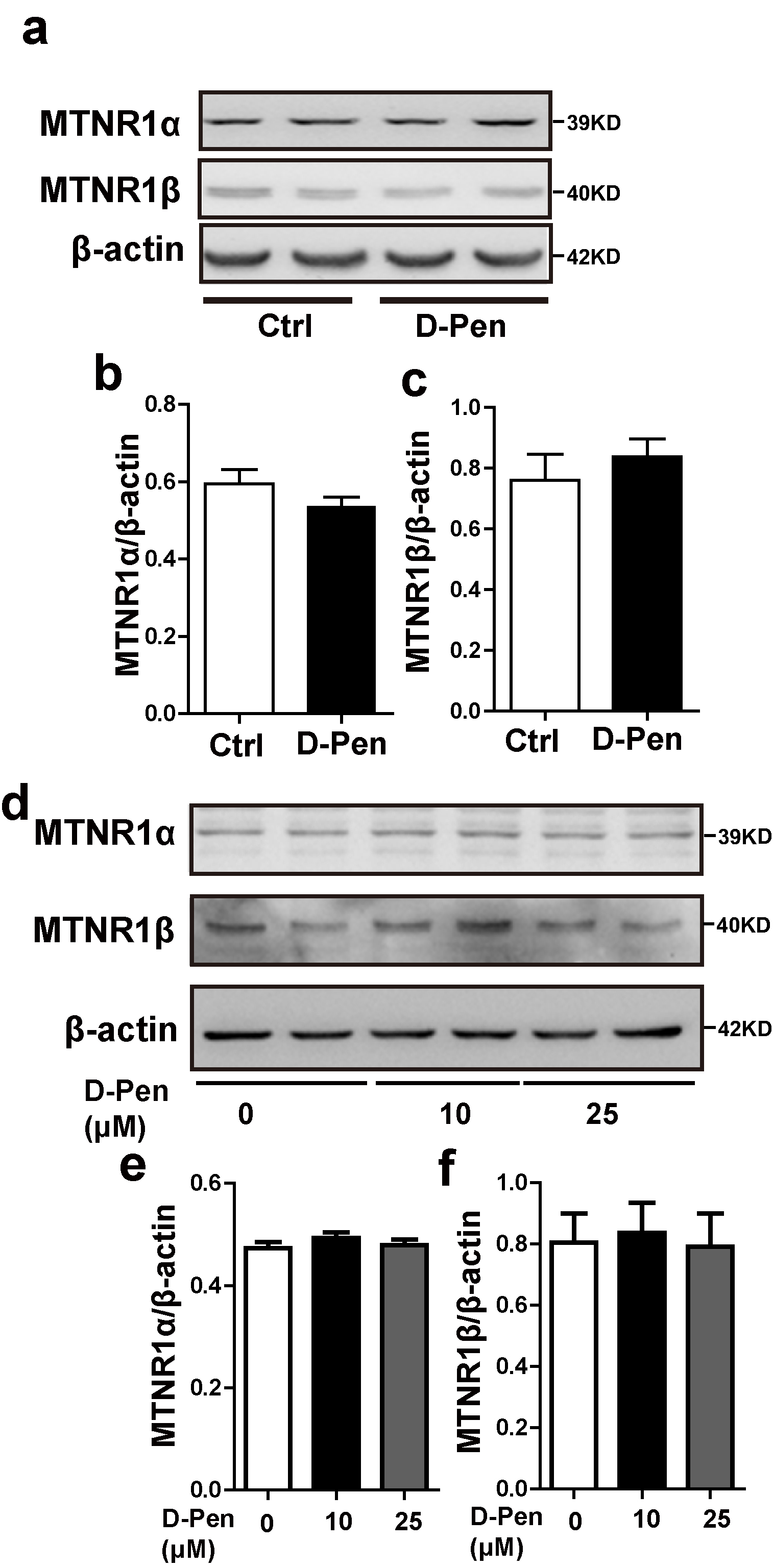

Supplement: Supplementary file 4 [file Image_4.JPEG]

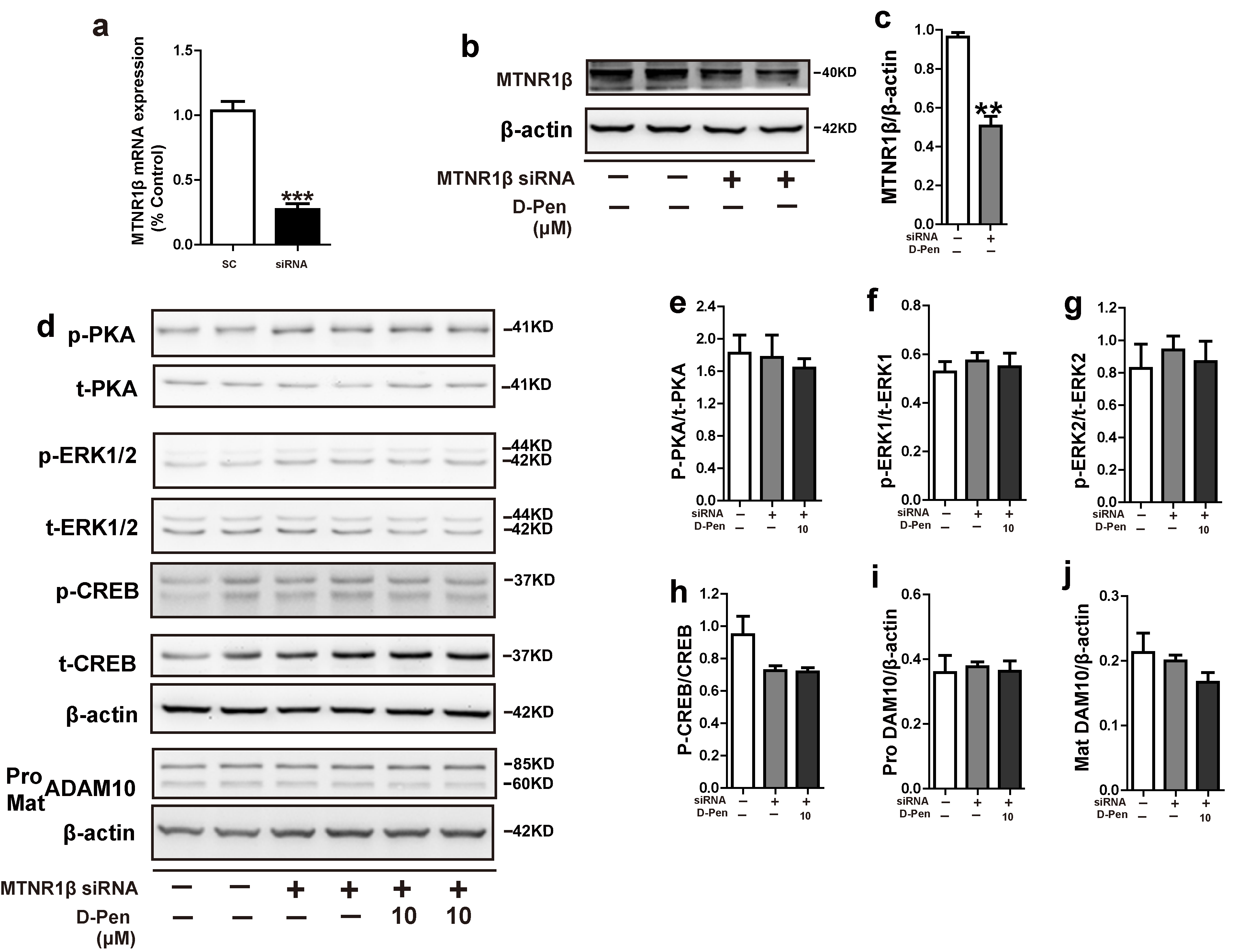

Supplement: Supplementary file 5 [file Image_5.JPEG]
