# Supplementary material for: Nasal Delivery of D-Penicillamine Hydrogel Upregulates a Disintegrin and Metalloprotease 10 Expression via Melatonin Receptor 1 in Alzheimer’s Disease Models
Source: Front Aging Neurosci. 2021 Apr 15;13:660249. doi: 10.3389/fnagi.2021.660249 (PMC8081912; doi:10.3389/fnagi.2021.660249)
Supplement: Supplementary file 6 [file Data_Sheet_1.docx]

**Supplementary Materials**

**Figure Legends**

**Figure S1. Synthesis of D-Pen-CS/β-GP hydrogel.** **(a)** Synthesis procedure of hydrogel. **(b)** The chemical structures of CS, β-GP and D-Pen. **(c)** Schematic diagram of hydrogel structure.

**Figure S2. Immunohistochemistry investigated APP/PS1 mice (4 month old) treated with PBS, CS/β-GP, D-Pen liquid, or D-Pen-CS/β-GP hydrogel for 3 month via intranasal administration. (a)** Aβ immunohistochemistry revealed SPs in the cerebral cortex in these groups. Bar = 30 μm. Representative Aβ plaques in coronal brain sections are shown in the black frame. Plaque numbers **(b)** and amyloid plaques size **(c)** in coronal sections were analyzed using ImageJ (n = 3). In the D-Pen hydrogel group, the number of Aβ-positive plaques decreased, and the plaques were smaller than those in the other three groups. No significant differences in the number of Aβ plaques were observed between the D-Pen liquid group/CS group and the PBS group. All values are presented as the mean ± standard error of the mean (SEM). Statistical significance in multiple comparisons was determined by one-way analysis of variance, * *p* < 0.05 compared to the vehicle control group, n = 5 of each group.

**Figure S3. D-Pen enhanced expression of ADAM10 and upregulated the expression of PKA-ERK-CREB pathway in primary neurons injured by Aβ.** Expressions of APP and APP-processing proteins were assayed by Western blot. Representative immunoblots **(a)** and quantification **(b-g)** of APP and APP-processing proteins expression. The expression of proteins was normalized by β-actin. D-Pen treatments increased the protein levels of mature ADAM10 in primary cortical neurons with Aβ injury. All values are presented as means ± SEM (**p* < 0.05 *vs* control group, #*p* < 0.05 *vs* Aβ group). Primary neurons were pre-treated for with Aβ, followed by incubation with D-Pen and Aβ as described above. **(h)** Representative immunoblots of p-PKA, PKA, p-ERK1/2, ERK1/2, p-CREB, and CREB. **(i-l)** Quantitative analysis for p-PKA/PKA, p-ERK1/2/ERK1/2 and p-CREB/CREB. All values are presented as means ± SEM. All values are presented as means ± SEM (**p* < 0.05 *vs* control group, #*p* < 0.05 *vs* Aβ group).

**Figure S4. MTNR1α and MTNR1β expressions were not changed after D-Pen treatments *in vivo* and *in vitro.*** **(a)** The cerebral cortices of mice from the control and D-Pen groups were lysed (n = 5), and quantitation of the proteins including MTNR1α **(b)** and MTNR1β **(c)** was detected by Western blot analysis. N2a-sw cells were treated with 10 or 25 μM of D-Pen in FBS-free DMEM for 24 h. Immunoblotting images **(d)** and quantification including MTNR1α **(e)** and MTNR1β **(f)** are shown in the figure. β-actin was used as an internal control. Data are represented as the mean ± standard error of the mean of at least three independent experiments. Statistical significance in multiple comparisons was determined by two-tailed Student’s t-test or by one-way analysis of variance.

**Figure S5. D-Pen has no effect on the regulation of MTNR1β signaling pathway. (a)** Down-regulation of *MTNR1β*mRNA expression by the recombinant plasmids, respectively. The mRNA expression level was detected by the real-time PCR assay. Immunoblotting images **(b)** and quantification **(c)** showed MTNR1β was downregulated in N2a-sw cells. Immunoblots **(d)** and quantification results showed p-PKA/PKA **(e)**, p-ERK/ERK **(f-g)**, p-CREB/CREB **(h)** and pro-ADAM10 **(i)** or mature ADAM10 **(j)** in N2a-sw cells with knockdown MTNR1β following which they were incubated with 10 μM D-Pen. Data are represented as the mean ± standard error of the mean of at least three independent experiments. Statistical significance in multiple comparisons was determined by one-way analysis of variance, **p* < 0.05 compared to the control group.
